# Biomimetic vesicles engineered from modified tumour cells act as personalized vaccines for post-surgical cancer immunotherapy

**DOI:** 10.1038/s41565-025-02113-w

**Published:** 2026-01-29

**Authors:** Pei Yu, Zhiwei Jin, Lulu Meng, Zhiqiang Shi, Meng Li, Jun Luo, Xiong Zhu, Lei Yang, Yong Yin, Chao Zhang, Lingyi Kong

**Affiliations:** 1 State Key Laboratory of Natural Medicines, Basic Medical Research Innovation Center for Anti-Cancer Drugs (Ministry of Education), Jiangsu Key Laboratory of Bioactive Natural Product Research, China Pharmaceutical University, Nanjing, China; 2 School of Engineering, China Pharmaceutical University, Nanjing, China

**Keywords:** Nanobiotechnology, Biomaterials

## Abstract

Surgical resection remains the primary treatment for most solid tumours, yet metastatic tumour cells remaining after surgery substantially contribute to cancer-related mortality and recurrence. Here we identify syntaxin 11 as a key regulator that enhances the expression of MHC I and co-stimulatory molecules CD80/CD86 on tumour cell membranes, enabling cancer cells to acquire dendritic-cell-like features. By overexpressing syntaxin 11 in autologous tumour cells obtained from surgical resections, we generated MHC I^high^/CD80^high^/CD86^high^ dendritic-cell-like cells. Utilizing the cell membranes of these modified cells, we engineered artificial dendritic-cell-like cell-derived vesicles as a personalized autologous nanovaccine for the immunotherapy of postoperative metastatic cancer. This nanovaccine substantially improves antigen delivery to lymphoid organs and enhances antigen presentation efficiency through tumour self-presentation, thereby disrupting traditional vaccine development paradigms. Our work provides a promising avenue for developing effective metastatic cancer immunotherapies and offers hope for personalized postoperative immunotherapy.

## Abstract (web summary)

Personalized nanovaccines derived from autologous tumour cells and engineered to display dendritic cell-like features enhance immune activation and prevent postoperative metastatic cancer in preclinical models.

## Main

Surgery remains the primary treatment for most solid tumours; however, residual metastatic cells after resection are major contributors to tumour recurrence and cancer-related mortality^1,2^. Cancer immunotherapy offers a promising strategy to eliminate these residual cells^3,4^, with the activation of CD8^+^ cytotoxic T lymphocytes (CTLs) being a central determinant of therapeutic efficacy. Personalized cancer vaccines, particularly dendritic cell (DC) vaccines loaded with autologous tumour lysates, can induce antigen-specific CTL responses^5–8^. Nevertheless, DCs activated by tumour lysates suffer from limited in vivo survival and inefficient migration to draining lymph nodes (dLNs), which restricts CTL priming^9–11^. Consequently, the objective response rate of current DC vaccines remains below 20% in clinical settings^5,12^, underscoring a critical need for improved postoperative immunotherapeutic strategies.

Artificial-cell-derived vesicles (ACDVs) have emerged as an alternative to living DC vaccines. By transferring natural membrane components onto synthetic nanocarriers, ACDVs can display functional peptide-MHC I complexes and co-stimulatory molecules, partially substituting endogenous antigen-presenting cell (APC) functions^13–15^. Compared with natural APCs, nanoscale ACDVs exhibit prolonged circulation and improved dLN accumulation, enabling the direct stimulation of CD8^+^ T cells^16^. Several studies have demonstrated the feasibility of this strategy, including DC membrane vesicles bearing p-MHC I and CD80/CD86 (ref. ^8^), lentivirally engineered tumour-derived vesicles^16^ and adenovirus-mediated reprogramming of tumour cells into APC-like cells^17,18^. However, a fundamental barrier to clinical translation remains: tumour neoantigens are highly patient specific and unpredictable^19,20^, making it difficult to generate truly personalized and rapidly deployable ACDVs for postoperative immunotherapy. Moreover, existing approaches to endow tumour cells with APC functionality are often complex, time-consuming or insufficiently effective in the postoperative metastatic setting.

**Fig. 1:**
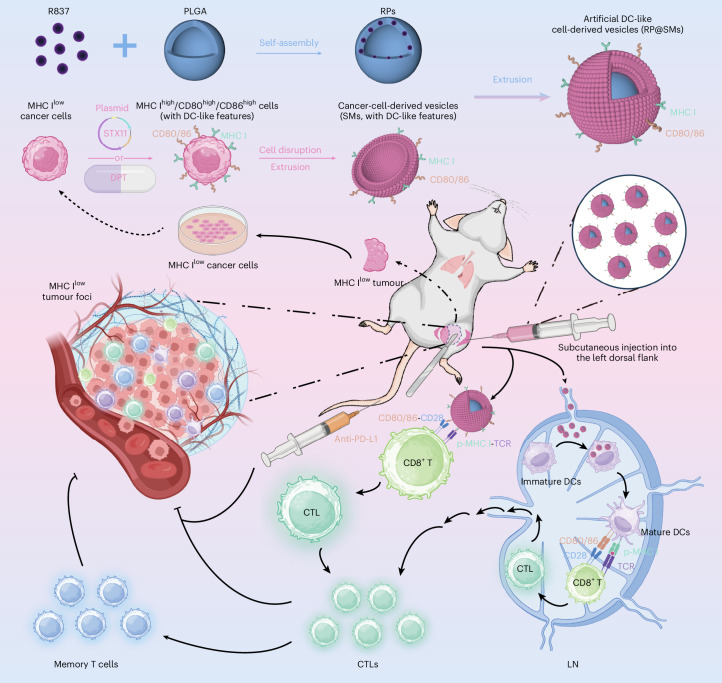
Schematic of the preparation of RP@SMs and their application as a personalized autologous vaccine for postoperative metastatic cancer immunotherapy. Surgically resected autologous tumour cells are transfected to overexpress STX11, yielding MHC I^high^/CD80^high^/CD86^high^ DC-like cells. The cell membranes of these modified cells are co-extruded with PLGA nanoparticles loaded with the immunoadjuvant imiquimod (R837) to produce RP@SMs. This personalized nanovaccine presents both p-MHC I and co-stimulatory molecules CD80/CD86 directly and indirectly to CD8^+^ T cells, stimulating a robust CTL response and, therefore, exerting antitumour effects against recurrence and metastasis. Figure created with BioRender.com. Source data

Here we identify syntaxin 11 (STX11), highly expressed in immune cells but low in tumour cells, as a regulator of tumour cell antigen-presenting capacity. We demonstrate that STX11 enhances the surface expression of MHC I and the co-stimulatory molecules CD80 and CD86, conferring DC-like features on tumour cells. By overexpressing STX11 in autologous tumour cells obtained from surgical resections, we generated MHC I^high^/CD80^high^/CD86^high^ DC-like tumour cells, whose membranes were used to construct artificial DC-like cell-derived vesicles (RP@SMs) as personalized nanovaccines for postoperative metastatic cancer immunotherapy (Fig. 1). To address the clinical need for rapid vaccine preparation after surgery, we further identify the natural compound deoxypodophyllotoxin (DPT) as a small-molecule alternative to plasmid-based STX11 overexpression that accelerates vaccine production and preserves potent antitumour efficacy. Together, this work establishes a strategy to directly reprogram autologous tumour cells into DC-like antigen-presenting platforms, overcoming key limitations in current DC and ACDV vaccines and filling a critical gap in personalized postoperative cancer immunotherapy^21,22^.

## STX11 confers DC traits on tumours

**Fig. 2:**
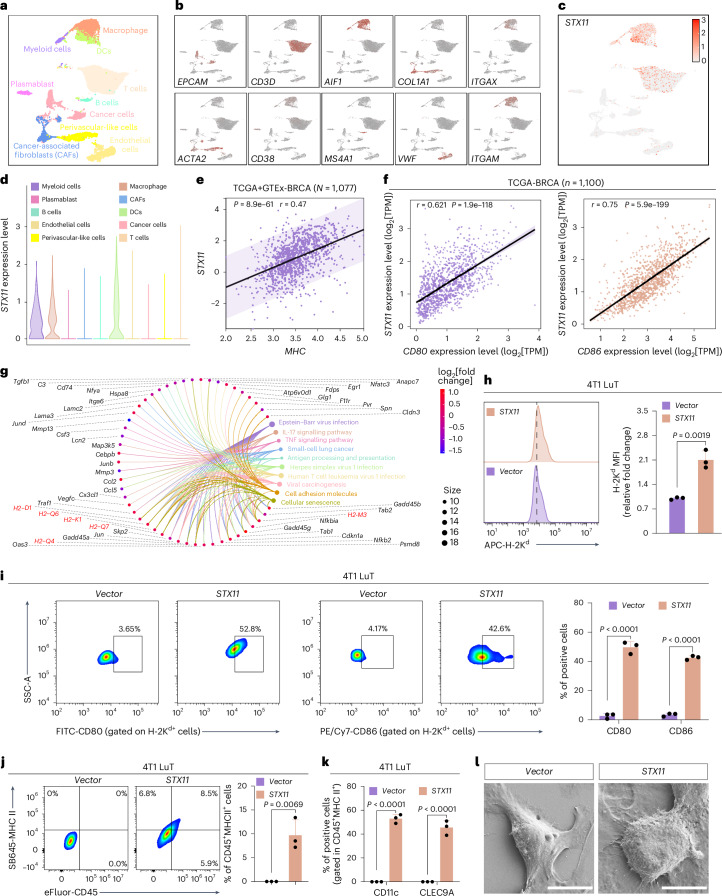
Characterization of STX11-overexpressing tumour cells. **a**, UMAP visualization of single-cell transcriptomic data from breast cancer patient tumours with annotated cell types. **b**, Expression of lineage-specific marker genes for cancer cells (*EPCAM*), T cells (*CD3D*), macrophages (*AIF1*), cancer-associated fibroblasts (*COL1A1*), DCs (*ITGAX*), perivascular-like cells (*ACTA2*), plasmablasts (*CD38*), B cells (*MS4A1*), endothelial cells (*VWF*) and myeloid cells (*ITGAM*). **c**, *STX11* expression across all cell clusters. **d**, Violin plots showing *STX11* expression levels in the indicated cell types. **e**, Correlation between *STX11* and *MHC* molecules in BRCA patients. **f**, Correlation between *STX11* and *CD80/86* in BRCA patients analysed using TIMER2.0. TPM, transcripts per million. **g**, CNet plot from KEGG enrichment analysis showing the top-ten significantly altered pathways and their associated differentially expressed genes in 4T1 LuT cells transfected with *STX11*. Genes with *P*_adjust_ < 0.05 and |log_2_[fold change]| > 0.3 are highlighted (*n* = 2 independent biological replicates). **h**, Flow cytometry analysis of surface MHC I levels in 4T1 LuT cells transfected with *STX11* or *Vector* (*n* = 3 independent biological replicates). MFI, mean fluorescence intensity. **i**, Percentages of CD80^+^MHC I^+^ and CD86^+^MHC I^+^ populations in *STX11*^−^ or *Vector*-transfected 4T1 LuT cells (*n* = 3 independent biological replicates). **j**, Flow cytometry analysis of the percentage of CD45^+^MHC II^+^ cells in 4T1 LuT cells at day 3 post-transfection with *STX11* or *Vector* (*n* = 3 independent biological replicates). **k**, Quantification of CD11c^+^ and CLEC9A^+^ cells within the gated CD45^+^MHC II^+^ population (*n* = 3 independent biological replicates). **l**, Scanning electron microscopy images of 4T1 LuT cells at day 3 post-transfection with *STX11* or *Vector* (*n* = 3 independent biological replicates). Scale bars, 10 μm. Data are presented as mean ± s.d. Statistical significance was assessed using an unpaired two-sided *t*-test with a confidence interval of 95%. Source data

STX11, a soluble NSF attachment protein receptor family member, critically regulates immune responses^23^. Pan-cancer transcriptomic analyses (TCGA and GTEx, 19 cancer types) revealed significantly elevated *STX11* expression in normal tissues versus tumours (Supplementary Fig. 1a). High *STX11* expression correlated with improved overall survival in metastatic breast cancer and melanoma (Supplementary Fig. 1b). Single-cell RNA sequencing of breast cancer tissues confirmed minimal *STX11* in tumour cells but robust expression in immune cells, particularly DCs (Fig. 2a–d). RNA sequencing of bone-marrow-derived DCs (BMDCs) transfected with *STX11* revealed an enrichment of immune response pathways, including antigen processing/presentation and cytokine–receptor interactions (Supplementary Fig. 2a,b). MHC I genes (*H2-K1*, *H2-D1* and *H2-Q6*) and co-stimulatory molecules (*CD80* and *CD86*) were upregulated (Supplementary Fig. 2c), whereas STX11 knockdown suppressed these effectors (Supplementary Fig. 2d,e). Flow cytometry validated that *STX11* overexpression enhanced BMDC maturation and surface MHC I expression (Supplementary Fig. 2f,g). Clinical correlation analyses demonstrated an inverse association between STX11 and breast cancer stage (Supplementary Fig. 3), suggesting its potential to confer DC-like features on tumour cells.

**Extended Data Fig. 1:**
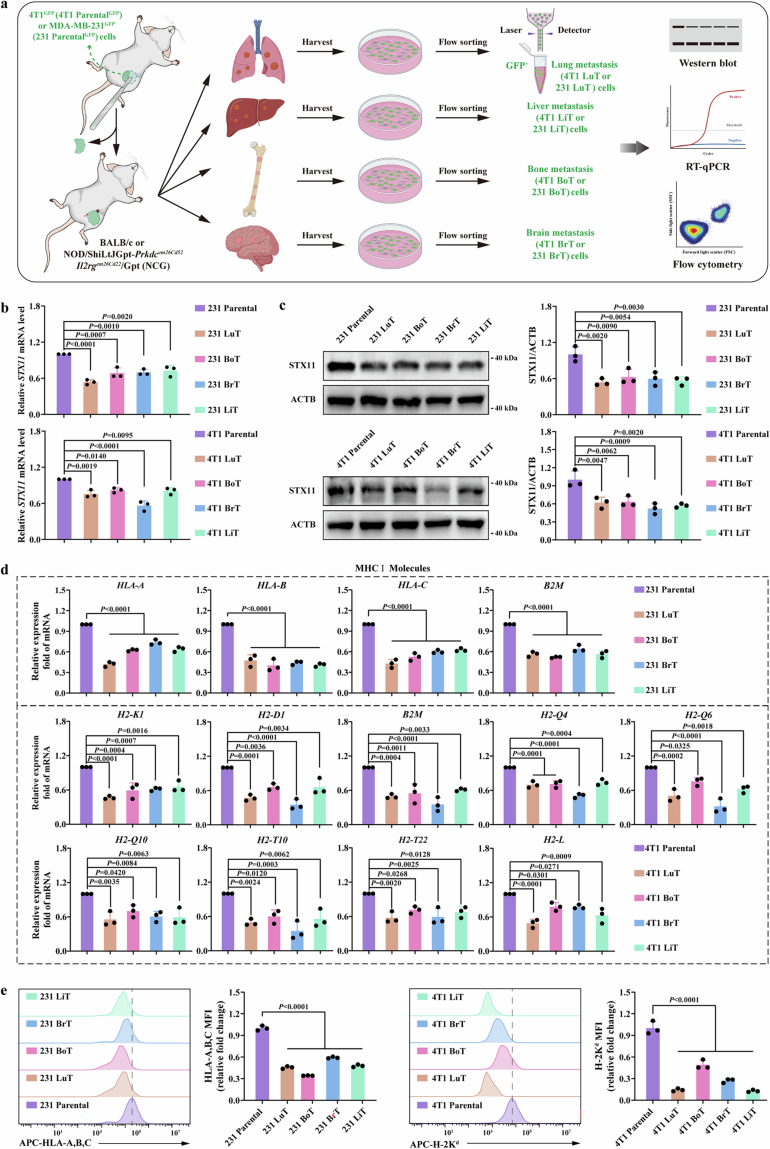
Low expression of STX11 and MHC I molecules in postoperative metastatic TNBC cells. **a**, Workflow for obtaining postoperative TNBC cells metastasized to the lungs, liver, bones, and brain. **b**, STX11 mRNA expression in parental and postoperative metastatic TNBC cells (*n* = 3 independent biological replicates). **c**, STX11 protein levels in parental and postoperative metastatic TNBC cells (*n* = 3 independent biological replicates). **d**, mRNA expression of MHC I molecules in parental and metastatic TNBC cells measured by RT-qPCR (*n* = 3 independent biological replicates). **e**, Flow-cytometry analysis of surface MHC I expression on parental and postoperative metastatic TNBC cells (*n* = 3 independent biological replicates). Data are presented as mean ± s.d. Statistical significance was determined using two-sided one-way ANOVA followed by Tukey’s multiple-comparisons test with a confidence interval of 95%. Panel **a** created with BioRender.com. Source data

To test this, we examined correlations between *STX11* expression and *MHC*/*CD80*/*CD86* levels across cancers. In breast cancer, *STX11* positively correlated with these markers (Fig. 2e,f and Supplementary Fig. 4a), and higher *STX11* expression was associated with increased immune infiltration (Supplementary Fig. 4b,c). Given that MHC I downregulation is a common immune evasion mechanism in metastatic tumours^24–26^, we explored whether STX11 could restore the antigen-presenting capacity in postoperative metastatic triple-negative breast cancer (TNBC) cells. Orthotopic TNBC models were generated using MDA-MB-231 or 4T1 cells, followed by ~90% primary tumour resection. Three weeks post-surgery, organ-specific metastatic subpopulations were isolated (Extended Data Fig. 1a). Compared with parental cells, these metastatic cells exhibited reduced STX11 and MHC I expression (Extended Data Fig. 1b–e). Focusing on lung metastases as the dominant cause of TNBC mortality^27^, *STX11* overexpression in 4T1 LuT cells enriched antigen presentation pathways and upregulated *MHC I*/*CD80*/*CD86* (Fig. 2g–i and Supplementary Figs. 5 and 6), whereas *STX11* knockdown suppressed MHC I (Supplementary Fig. 7). In particular, *STX11*-overexpressing 4T1 LuT cells generated a CD45^+^MHC II^+^ subpopulation^20^, with 52.93 ± 3.65% expressing CD11c and 45.47 ± 6.02% expressing CLEC9A, and displayed dendritic morphology (Fig. 2j–l). Together, these findings demonstrate that STX11 endows tumour cells with DC-like features by upregulating MHC I and co-stimulatory molecules, thereby providing a basis for tumour-derived artificial DC vaccine development.

## Engineering and profiling of biomimetic vesicles

**Fig. 3:**
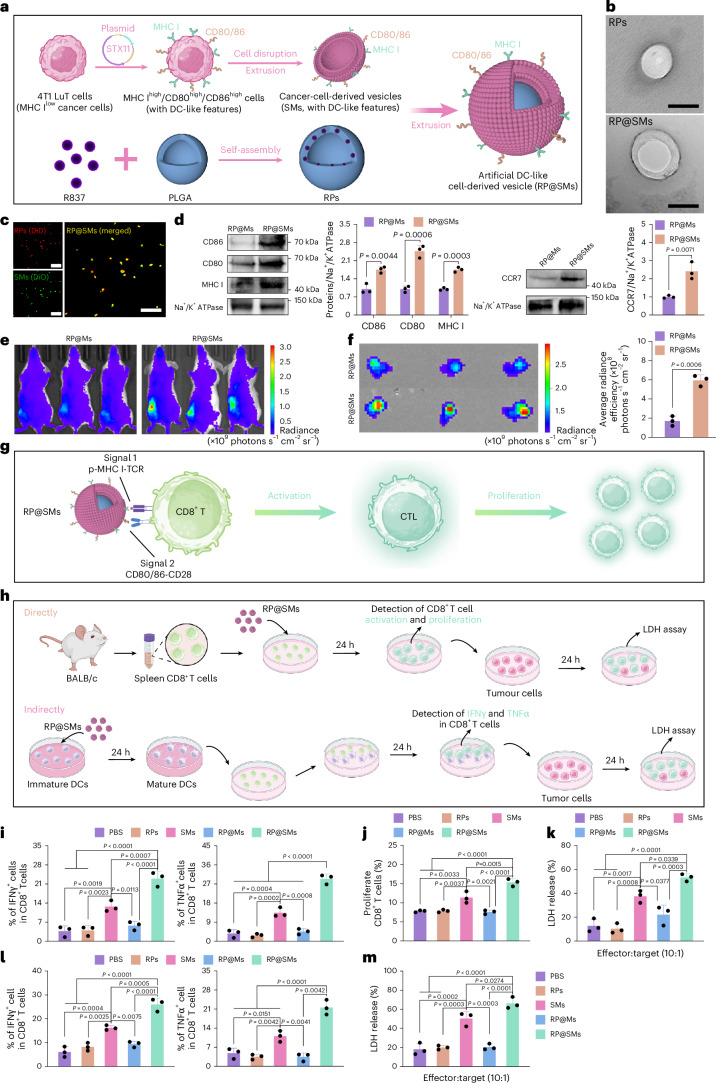
Preparation, characterization, CD8^+^ T cell activation and LN targeting of RP@SMs. **a**, Schematic of RP@SMs nanovaccine preparation. **b**, Transmission electron microscopy images of RPs and RP@SMs (*n* = 3 independent biological replicates). Scale bars, 200 nm. **c**, Confocal microscopy images of RP@SMs, with RPs labelled by DiD (red) and SMs labelled by DiO (green) (*n* = 3 independent biological replicates). Scale bars, 4 μm. **d**, Western blot analysis of CD80, CD86, MHC I and CCR7 expressions in RP@Ms (RPs co-extruded with membranes derived from 4T1 LuT cells transfected with an empty vector) and RP@SMs (*n* = 3 independent biological replicates). **e**,**f**, In vivo bioluminescence imaging (**e**) and fluorescent imaging of inguinal dLNs (**f**) after the subcutaneous injection of DiD-labelled RP@Ms or RP@SMs (*n* = 3 independent biological replicates). **g**, Schematic showing CD8^+^ T cell activation and proliferation by RP@SMs. **h**, Schematic of direct and indirect T cell activation assays corresponding to the data in **i**–**m**. **i**,**j**, Quantification of the percentages of IFNγ^+^CD8^+^ and TNFα^+^CD8^+^ T cells (**i**) and proliferating CD8^+^ T cells from mouse spleens (**j**) (*n* = 3 independent biological replicates). **k**, In vitro cytotoxicity of splenic T cells against 4T1 LuT cells after 24-h incubation with various nanoparticles (*n* = 3 independent biological replicates). **l**, Quantification of IFNγ^+^CD8^+^ and TNFα^+^CD8^+^ T cells in the DC-to-T co-culture system after 24-h incubation of BMDCs (pretreated with different nanoparticles) and splenocytes at a 1:10 ratio (*n* = 3 independent biological replicates). **m**, In vitro assessment of the cytotoxic activity of splenocytes, stimulated by BMDCs pretreated with various nanoparticles, against 4T1 LuT cells after 24-h co-culture at an effector-to-target ratio of 10:1 (*n* = 3 independent biological replicates). Data are presented as mean ± s.d. Statistical significance was determined using a one-way ANOVA with Tukey’s multiple comparisons test or an unpaired two-sided *t*-test with a confidence interval of 95%. Panels **a**, **g** and **h** created with BioRender.com. Source data

To improve antigen presentation, we engineered artificial DC-like vesicles that mimic endogenous APCs by displaying MHC molecules and co-stimulatory signals^16,28,29^. Building on our observation that *STX11* overexpression upregulates MHC I, CD80 and CD86 in TNBC cells, we derived vesicles (SMs) from these engineered cells and fused them with poly(lactic-*co*-glycolic acid) (PLGA) nanoparticles carrying the TLR7 agonist R837 (RPs), generating RP@SMs (Fig. 3a). Transmission electron microscopy, confocal imaging and physicochemical analyses confirmed efficient encapsulation and stable nanostructure (Fig. 3b,c and Supplementary Fig. 8a–c). Stability was improved when SMs were used in excess, and RP@SMs showed minimal size variation in serum over 24 h (Supplementary Fig. 8d,e). R837 release accelerated under acidic conditions (Supplementary Fig. 8f), consistent with endosomal uptake and cytoplasmic antigen release^30^. Immunoblotting-verified preservation of MHC I, CD80, CD86 and CCR7 levels were 2.4-fold higher than in control vesicles (RP@Ms), suggesting enhanced lymph node (LN) homing (Fig. 3d and Supplementary Fig. 8g).

Effective in vivo targeting requires LN accumulation. DiD-labelled RP@SMs injected subcutaneously accumulated predominantly in dLNs within 12 h, with minimal off-target distribution (Fig. 3e,f and Supplementary Fig. 9a,b). By contrast, RP@Ms showed weaker LN fluorescence. This preferential accumulation may arise from conserved lymph-homing receptors (for example, CCR7) on SMs^8,31^, enabling receptor–ligand interactions (p-MHC I:TCR, CD80/86:CD28) for T cell engagement.

Biosafety is crucial for the clinical translation of RP@SMs. Comprehensive biosafety evaluation showed that plasma biochemical parameters in RP@SMs-treated mice remained within normal physiological ranges (Supplementary Fig. 10a). Additionally, haematoxylin and eosin (H&E) staining of major organs showed no significant pathological changes (Supplementary Fig. 10b). These results indicate that the RP@SMs nanovaccine demonstrates good biocompatibility and biosafety for potential therapeutic use.

## RP@SMs activate CD8^+^ T cells via dual mechanisms

**Extended Data Fig. 2:**
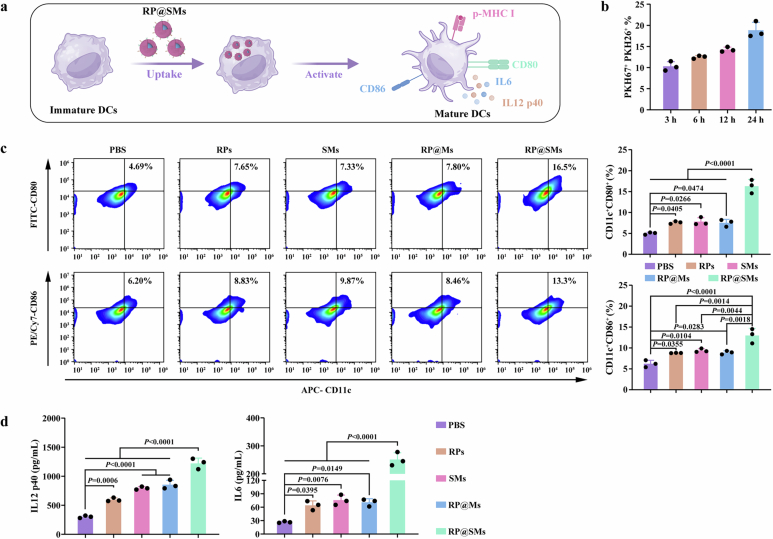
Activation of DCs by RP@SMs. **a**, Schematic illustrating the uptake and activation of BMDCs by RP@SMs. **b**, Time-course flow-cytometry analysis of BMDC uptake of RP@SMs (PKH26-labelled RPs and PKH67-labelled SMs) (*n* = 3 independent biological replicates). **c**, Representative flow-cytometry plots and quantification of CD11c + CD80+ and CD11c + CD86+ BMDCs after 24-hour incubation with different nanoparticles (*n* = 3 independent biological replicates). **d**, IL-12 p40 and IL-6 levels in culture supernatants of BMDCs after the indicated treatments (*n* = 3 independent biological replicates). Data are presented as mean ± s.d. Statistical significance was assessed using two-sided one-way ANOVA followed by Tukey’s multiple-comparisons test with a confidence interval of 95%. Panel **a** created with BioRender.com. Source data

To assess the immunogenic potential of RP@SMs, we first evaluated their capacity to activate DCs (Extended Data Fig. 2a), as the internalization of tumour-associated antigens by DCs is a critical step in antigen processing and presentation^32^. Flow cytometry revealed a time-dependent increase in the BMDC uptake of RP@SMs, reaching 18.8 ± 1.89% at 24 h (Extended Data Fig. 2b and Supplementary Fig. 11a,b). DC activation analysis demonstrated significantly increased CD80^+^ and CD86^+^ BMDCs in RPs and RP@Ms groups versus phosphate-buffered saline (PBS), with RP@SMs showing superior activation (Extended Data Fig. 2c and Supplementary Fig. 11c). This increased activation enabled DCs to present antigens more efficiently to T cells, initiating a robust immune response capable of targeting and eliminating tumour cells^33^. Supporting these findings, proinflammatory cytokine release (IL-6 and IL-12 p40) from mature BMDCs was significantly elevated (Extended Data Fig. 2d), suggesting that R837 enhances DC activation, and SMs further amplify antigen presentation.

We next examined direct CD8^+^ T cell activation via membrane-bound signals (p-MHC I:TCR and CD80/86:CD28 interactions; Fig. 3g). Splenic CD8^+^ T cells exposed to RP@SMs exhibited significantly higher TNFα^+^ and IFNγ^+^ frequencies versus controls and enhanced proliferation (Fig. 3h–j and Supplementary Fig. 12a–d). Cytotoxicity assays confirmed superior tumour cell lysis by RP@SMs-primed T cells (Fig. 3k). To evaluate indirect DC-mediated cross-presentation, BMDCs preincubated with RP@SMs were co-cultured with naive CD8^+^ T cells (Fig. 3h). This indirect priming similarly enhanced TNFα^+^CD8^+^ and IFNγ^+^CD8^+^ T cells, with potent tumour-killing capacity (Fig. 3l,m and Supplementary Fig. 13e). Together, these results demonstrate that RP@SMs, endowed with surface functional proteins characteristic of mature DCs, can activate CD8^+^ T cells in vitro through both direct antigen presentation and DC-mediated cross-priming pathways.

## RP@SMs elicit tumour-specific immune responses in vivo

**Fig. 4:**
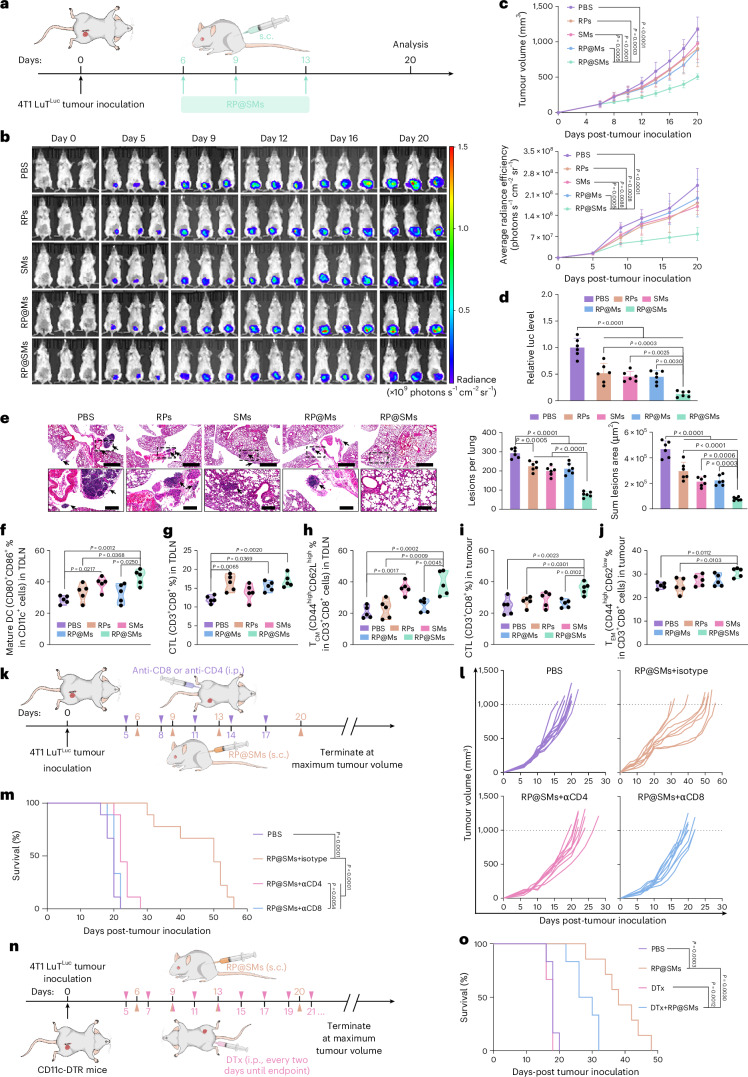
In vivo antitumour efficacy and immune responses induced by RP@SMs in postoperative metastatic tumour recurrence model. **a**, Schematic of tumour inoculation, treatment schedule and analysis time points. **b**, In vivo bioluminescence imaging of 4T1 LuT^Luc^-tumour-bearing mice on days 0, 5, 9, 16 and 20 post-treatment, with the quantification of fluorescence intensity at day 20 across treatment groups (*n* = 6 mice per group). **c**, Tumour growth curves of 4T1 LuT^Luc^-tumour-bearing mice following different treatments (*n* = 10 mice per group). **d**, Relative fluorescence intensity of the lungs at day 20 across treatment groups (*n* = 6 mice per group). **e**, H&E staining and the quantification of lung metastases in different treatment groups (*n* = 6 mice per group). Scale bars, 1 mm and 200 μm. **f**–**h**, Flow cytometric quantification of immune cell populations in TDLNs (*n* = 5 mice per group): mature DCs (CD11c^+^CD80^+^CD86^+^) (**f**); CTLs (CD3^+^CD8^+^) (**g**); central memory CD8^+^ T (T_CM_, CD3^+^CD8^+^CD44^high^CD62L^high^) cells (**h**). **i**,**j**, Quantification of tumour-infiltrating lymphocyte subsets (*n* = 5 mice per group): CTLs (CD3^+^CD8^+^) (**i**); effector memory CD8^+^ T (T_EM_, CD3^+^CD8^+^CD44^high^CD62L^low^) cells (**j**). **k**, Experimental timeline assessing the role of CD4^+^ and CD8^+^ T cells in RP@SMs-induced antitumour immunity using depleting antibodies. **l**, Individual tumour growth trajectories for each treatment group (*n* = 9 mice per group). **m**, Kaplan–Meier survival analysis (*n* = 9 mice per group). **n**, Experimental design evaluating the role of DCs using DTx-mediated depletion in CD11c-DTR mice, combined with RP@SMs vaccination. i.p., intraperitoneal; s.c., subcutaneous. **o**, Survival curves for each treatment group (*n* = 6–7 mice per group). Data are presented as mean ± s.d. Statistical significance was determined using a one-way ANOVA with Tukey’s multiple comparisons test or a log-rank (Mantel–Cox) test with a confidence interval of 95%. Panels **a**, **k** and **n** created with BioRender.com. Source data

An effective cancer vaccine should elicit tumour-specific immunity to prevent recurrence or metastasis^34^, particularly in TNBC, where surgery is followed by immunotherapy to eliminate residual cells^35,36^. Postoperative 4T1 LuT metastases grew faster and reduced survival compared with parental 4T1 tumours (Supplementary Fig. 13). To model postoperative recurrence, we established a 4T1-LuT-derived metastatic breast cancer model (Fig. 4a). Mice with ~100-mm^3^ tumours were randomized into five groups and treated with PBS, RPs, SMs, RP@Ms or RP@SMs. Bioluminescence imaging showed slower tumour progression and stable body weight in RP@SMs-treated mice, which suppressed tumour growth by 57% and effectively prevented lung metastases (Fig. 4b–e and Supplementary Fig. 14a,b).

To confirm that RP@SMs induced a tumour-specific immune response, we replicated the experiment and sacrificed the mice on day 20 to collect tumour DLNs (TDLNs), spleens and tumours. TDLNs, as key immunological reservoirs, play a critical role in reactivating antitumour immunity and preventing metastasis^37,38^. Flow cytometry analysis revealed a significant increase in mature DCs and CTLs in the RP@SMs group (Fig. 4f,g and Supplementary Fig. 15a,b), indicating enhanced DC-mediated antigen presentation and a robust tumour-specific CTL response. Moreover, after RP@SMs vaccination, naive CD8^+^ T (T_naive_) cells and central memory CD8^+^ T (T_CM_) cells in the TDLNs increased by 74% and 90%, respectively, whereas effector memory CD8^+^ T (T_EM_) cells decreased by 65% (Fig. 4h and Supplementary Fig. 15c,d), suggesting that RP@SMs promoted T_CM_ cell accumulation in TDLNs, thereby enhancing antigen presentation and immune memory^39^. The spleen, an essential immune organ with strong immunological memory functions, also showed significant activation^40,41^. Flow cytometry revealed higher proportions of CTLs and T_EM_ cells in the spleens of RP@SMs-treated mice compared with the RPs and PBS groups (Supplementary Fig. 16). Similarly, in the tumour tissue, RP@SMs treatment significantly increased the proportions of CTLs and T_EM_ cells (Fig. 4i,j and Supplementary Fig. 17). These findings collectively demonstrate that RP@SMs vaccination activated robust immune responses across TDLNs, spleen and tumour tissues, contributing to effective tumour growth suppression and metastasis inhibition.

To delineate immune cell contributions, we depleted CD4^+^ or CD8^+^ T cells using neutralizing antibodies (Fig. 4k). Ablation of either subset impaired tumour control and survival, with CD8^+^ T cell depletion nearly abolishing antitumour activity (Fig. 4l,m), highlighting their non-redundant role. In parallel, using CD11c-DTR mice^42^, we showed that diphtheria toxin (DTx)-mediated DC depletion significantly reduced but did not completely eliminate the therapeutic effect of RP@SMs (Fig. 4n,o and Supplementary Figs. 18 and 19). These findings suggest that RP@SMs mediate potent antitumour activity through the synergy of DC-dependent antigen presentation and DC-independent mechanisms.

## RP@SMs with checkpoint blockade combat metastasis

**Fig. 5:**
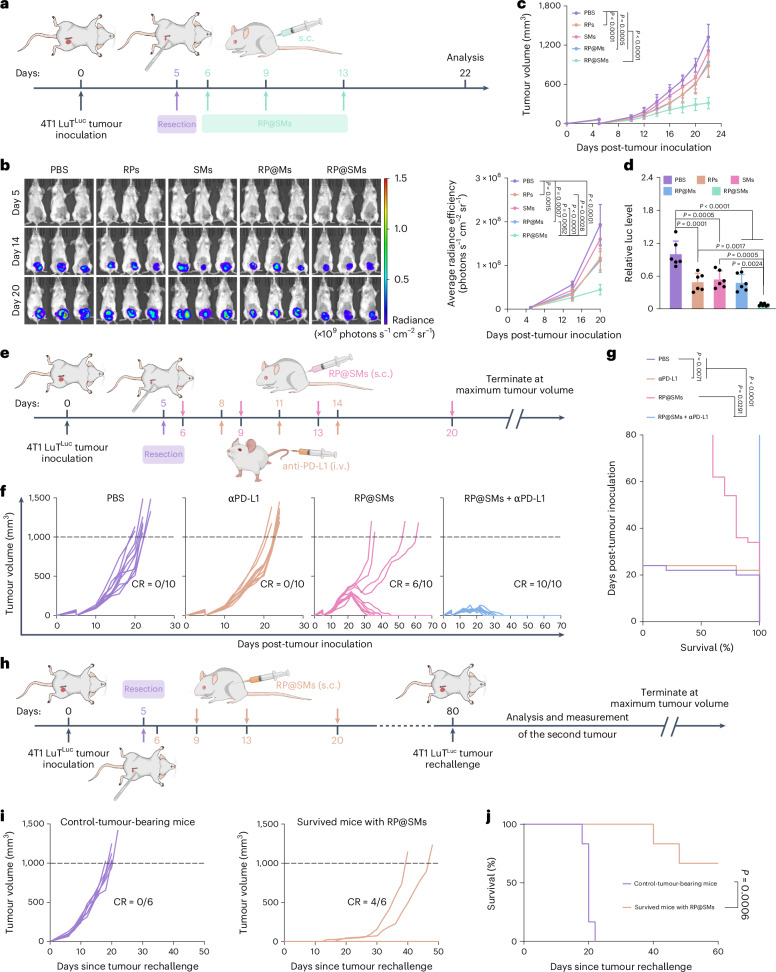
Therapeutic efficacy of the RP@SMs nanovaccine and its combination with anti-PD-L1 in postoperative metastatic TNBC. **a**, Schematic of partial tumour resection, vaccination schedule and analysis timeline. **b**, In vivo bioluminescence images and the quantification of fluorescence intensity in 4T1 LuT^Luc^-tumour-bearing mice at days 5, 14 and 20 across treatment groups (*n* = 6 mice per group). **c**, Tumour growth curves following different treatments (*n* = 10 mice per group). **d**, Relative fluorescence intensity in the lungs at day 20 across treatment groups (*n* = 6 mice per group). **e**, Schematic of the treatment protocol combining RP@SMs with anti-PD-L1 following tumour resection. i.v., intravenous. **f**, Individual tumour growth curves across treatment groups (*n* = 10 mice per group). CR, complete tumour regression. **g**, Survival curves for each treatment group (*n* = 10 mice per group). **h**, Schematic of tumour resection, RP@SMs vaccination and rechallenge with 4T1 LuT cells on day 80 to assess vaccine-induced immunological memory. **i**, Tumour growth curves after rechallenging (*n* = 6 mice per group). **j**, Corresponding survival curves following tumour rechallenge (*n* = 6 mice per group). Data are presented as mean ± s.d. Statistical significance was determined using a one-way ANOVA with Tukey’s multiple comparisons test or a log-rank (Mantel–Cox) test with a confidence interval of 95%. Panels **a**, **e** and **h** created with BioRender.com. Source data

To evaluate the therapeutic potential of RP@SMs against postoperative metastasis, we performed incomplete resection, leaving ~10% tumour mass when the tumours reached ~60 mm^3^ (Fig. 5a). Mice received RP@SMs immunization on postoperative days 1, 4 and 8. RP@SMs monotherapy achieved a 76% inhibition rate, outperforming other groups (Fig. 5b,c). In vivo imaging showed no lung fluorescence in RP@SMs-treated mice (Fig. 5d and Supplementary Fig. 20a), and histology confirmed markedly fewer metastatic foci, even lower than those in the non-resection model (Fig. 4e and Supplementary Fig. 20b), indicating strong synergy with surgery.

Given that PD-1/PD-L1 immune checkpoint blockade is a standard therapy for TNBC and TNBC cells often display elevated PD-L1 expression^43^, we analysed PD-L1 on tumour cells and PD-1 on CD8^+^ tumour-infiltrating lymphocytes. RP@SMs significantly reduced PD-1^+^CD8^+^ tumour-infiltrating lymphocytes without altering tumour PD-L1 (Supplementary Fig. 21), suggesting synergy potential with PD-L1 blockade. Accordingly, we tested RP@SMs plus anti-PD-L1 in orthotopic breast cancer (Fig. 5e). Although anti-PD-L1 monotherapy showed limited efficacy, combination with RP@SMs demonstrated significant synergistic antitumour activity. All PBS- and anti-PD-L1-treated mice succumbed by day 25, whereas RP@SMs monotherapy yielded 60% survival and the combination achieved 100% survival at day 80 (Fig. 5f,g), demonstrating potent and durable antitumour immunity.

To assess immune memory, tumour-free mice surviving RP@SMs treatment underwent secondary challenge with 4T1 LuT cells on day 80. Specifically, an equal number of 4T1 LuT tumour cells were reinoculated into the mammary fat pad of these mice, with age-matched, treatment-naive mice serving as controls (Fig. 5h). RP@SMs-pretreated mice displayed sustained tumour suppression and extended survival versus controls (Fig. 5i,j), confirming that RP@SMs act as artificial DC-like vesicle nanovaccines capable of inducing durable immune memory.

## A personalized autologous vaccine for cancer therapy

**Fig. 6:**
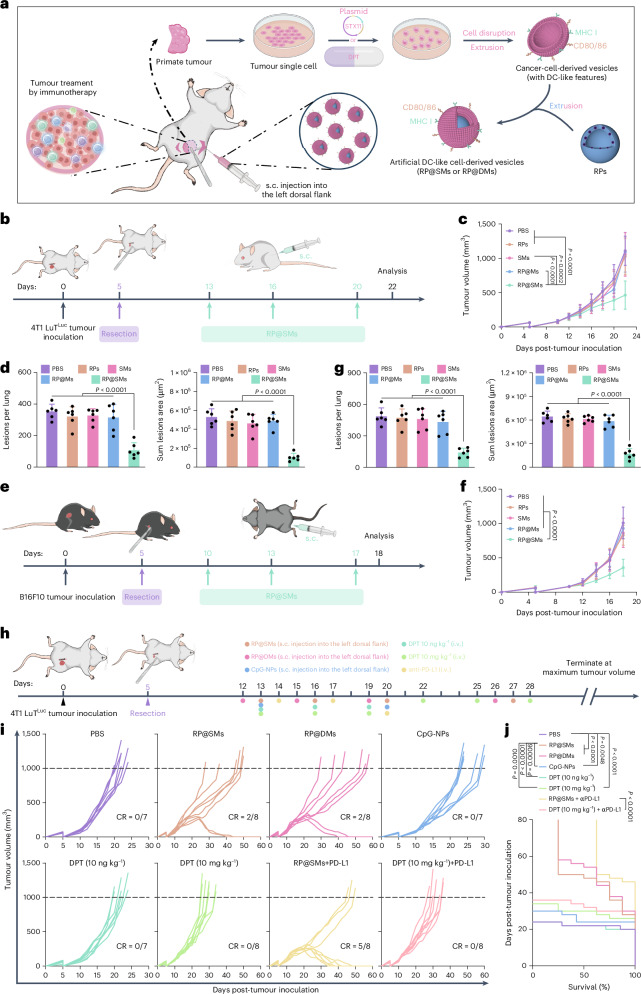
Personalized autologous vaccine for postoperative metastatic tumour therapy. **a**, Schematic of RP@SMs or RP@DMs fabrication using tumour cell membranes derived from surgically resected tumours. **b**,**e**, Schematic of partial tumour resection, personalized vaccination schedule and analysis timeline. **c**,**f**, Tumour growth curves in different treatment groups (*n* = 9–10 mice per group). **d**,**g**, Quantification of pulmonary metastases across treatment groups (*n* = 6 mice per group). **h**, Schematic of the timeline of personalized vaccination and additional therapeutic strategies in postoperative tumour-bearing mice. **i**, Individual tumour growth trajectories per treatment group (*n* = 7–8 mice per group). **j**, Kaplan–Meier survival curves for each treatment group (*n* = 7–8 mice per group). Data are presented as mean ± s.d. Statistical significance was determined using a one-way ANOVA with Tukey’s multiple comparisons test or a log-rank (Mantel–Cox) test with a confidence interval of 95%. Panels **a**, **b**, **e** and **h** created with BioRender.com. Source data

Human tumour mutations are highly patient specific^44^. To address this variability, we designed a clinically relevant study by isolating cancer cells from surgically resected autologous tumours (Fig. 6a). Specifically, 4T1 LuT^Luc^ cells were injected into the left mammary fat pad of BALB/c mice, and approximately 90% of the tumour was resected after 5 days. Excised tissue was processed into single-cell suspension and transfected with *STX11* plasmids, and cell-membrane-derived vesicles were co-extruded with RPs to generate personalized nanovaccines (Fig. 6a,b). After subcutaneous administration, tumour progression was monitored. Although preparation time allowed tumours to reach ~200 mm^3^ before vaccination, RP@SMs significantly delayed tumour growth and metastasis (Fig. 6c,d).

To assess broad applicability, we extended the evaluation to metastatic B16F10 melanoma (MHC I^low^) models^45,46^. Similar to the 4T1 LuT model, personalized RP@SMs significantly inhibited tumour growth and metastasis (Fig. 6e–g). No significant impact on body weight was observed throughout treatment (Supplementary Fig. 22), demonstrating safety and efficacy across tumour types. To further validate antigen specificity, 4T1-LuT-tumour-bearing mice received RP@SMs derived from allogeneic B16F10 cells (Supplementary Fig. 23a). These heterologous RP@SMs failed to inhibit tumour growth or improve survival (Supplementary Fig. 23b–d), confirming that RP@SM-induced antitumour immunity is strictly antigen specific.

## Accelerated preparation of personalized vaccine using DPT

The development of personalized nanovaccines is often limited by time-consuming plasmid transfection, delaying timely intervention. To address this, we screened natural compounds regulating STX11 and identified DPT as a candidate that dose dependently increases STX11 protein without significant cytotoxicity (Supplementary Fig. 24a–c). DPT substitutes for STX11 plasmids and markedly shortens vaccine preparation. We evaluated DPT at 6 nM and 16 nM in 4T1 LuT and 231 LuT TNBC cells; subsequent studies used these doses. Reverse transcription quantitative polymerase chain reaction (RT-qPCR) confirmed DPT-induced *STX11* upregulation (Supplementary Fig. 24d). DPT also elevated *MHC I* gene expression, surface MHC I protein (Supplementary Fig. 25) and CD80^+^/CD86^+^ cells (Supplementary Fig. 26), indicating that DPT-based artificial DC-like vesicles (RP@DMs) enhance immunogenicity and promote tumour-specific responses.

We next assessed RP@DMs in a postoperative metastatic breast cancer model (Supplementary Fig. 27a). RP@DMs markedly inhibited tumour growth and metastasis (Supplementary Fig. 27b,c). Flow cytometry showed increased mature DCs, CTLs and T_CM_ cells in TDLNs (Supplementary Fig. 27d–f), with elevated CTLs and T_EM_ cells in spleens and tumours (Supplementary Fig. 27g–j), demonstrating durable antitumour memory with reduced preparation time.

To confirm antigen specificity, tumour cells from autologous tissue were treated with *STX11* plasmids (RP@SMs) or low-dose DPT (10 ng kg^−1^; RP@DMs) to generate personalized vaccines (Fig. 6h). PBS- and low-dose-DPT-treated mice succumbed within 25 days. Although CpG-NPs or high-dose DPT delayed progression, all mice succumbed by day 36. In particular, RP@SMs and RP@DMs showed comparable efficacy, with 25% survival beyond 80 days. RP@SMs plus anti-PD-L1 achieved complete response and long-term survival (>80 days) in 5/8 mice (Fig. 6i,j), indicating that RP@DM efficacy derives primarily from antigen-specific immunity rather than DPT cytotoxicity.

## Conclusions

This study introduces a personalized nanovaccine platform in which autologous tumour cells are reprogrammed into DC-like antigen-presenting units through STX11 induction and subsequently converted into artificial DC-like vesicles for postoperative cancer immunotherapy. By preserving the full repertoire of patient-specific tumour antigens and incorporating defined co-stimulatory signals, RP@SMs circumvent the dependency on exogenous antigen identification and demonstrate potent, antigen-restricted CD8^+^ T cell activation and antitumour efficacy in multiple postoperative metastatic models. The DPT-based rapid reprogramming strategy further improves clinical feasibility by substantially shortening vaccine preparation time without compromising therapeutic performance.

However, several challenges must be addressed before clinical translation. Human tumours exhibit far greater genetic and phenotypic heterogeneity than murine models, which may influence STX11 responsiveness and membrane vesicle immunogenicity. In addition, autologous vesicle-based vaccines face practical constraints in patient-to-patient variability, real-time production logistics and regulatory quality control. Furthermore, although synergy with PD-L1 blockade was evident, the immunological window for maximum benefit after surgery remains undefined and may differ across tumour types and immunological backgrounds.

Future efforts should prioritize validation in patient-derived tumour tissues; development of closed, good-manufacturing-practice-compatible vesicle manufacturing systems; and systematic optimization of perioperative combination regimens with immune checkpoint inhibitors. Addressing these translational barriers will determine whether artificial DC-like vesicle vaccines can evolve from an individualized experimental strategy into a clinically deployable postoperative immunotherapy.

## Methods

### Cell lines and lentiviral infection

Human breast cancer cell line MDA-MB-231 (TCHu227), mouse breast cancer cell line 4T1 (TCM32) and mouse melanoma cell line B16F10 (TCM36) were sourced from the Cell Bank of the Shanghai Institute of Life Sciences. 4T1 and B16F10 cells were cultured in RPMI 1640 medium, whereas MDA-MB-231 cells were maintained in Dulbecco’s modified Eagle’s medium, both supplemented with 10% fetal bovine serum and 1% penicillin–streptomycin under standard conditions (37 °C, 5% CO_2_). Regular mycoplasma testing confirmed the absence of contamination.

For stable gene expression, cells at approximately 40% confluency were transduced with HBLV-GFP-Puro or HBLV-Luc-Puro lentiviral vectors (Hanbio Biotechnology) in the presence of 10 μg ml^−1^ polybrene to enhance infection efficiency. After 72 h, cells were selected with puromycin-containing medium to establish stable cell lines.

### Flow cytometric sorting of metastatic tumour cells

All animal experiments were reviewed and approved by the Animal Ethics Committee of China Pharmaceutical University (approval number 2021-01-021) and conducted in compliance with the approved maximum tumour burden limit of 2,000 mm^3^. Mice were housed under specific pathogen-free conditions with a 12 h light/dark cycle at 25 ± 1 °C and 50%–80% humidity, with ad libitum access to food and water. All mice were 6–8 weeks old at the start of experiments, and unless otherwise specified, female mice were used throughout. Animal handling was performed by personnel certified under the Jiangsu Provincial Experimental Animal Professional Skills Training Program (certification number 2162345).

Female BALB/c or NCG mice (GemPharmatech) were orthotopically injected with 5 × 10^5^ 4T1 Parental^GFP^ or 231 Parental^GFP^ cells into the left mammary fat pad. On day 10 post-inoculation, approximately 90% of the primary tumour was surgically resected. Three weeks after surgery, lungs, bones, brain and liver were harvested for analysis.

Tissues were enzymatically digested: lungs with type I collagenase and hyaluronidase (Sigma-Aldrich); bones by flushing femurs and tibias; brain with papain; liver with type IV collagenase, hyaluronidase and DNase I (Sigma-Aldrich). Following red blood cell (RBC) lysis and PBS washes, single-cell suspensions were subjected to flow cytometric sorting to isolate GFP-positive metastatic tumour cells from each organ (4T1 or 231 LuT^GFP^, BoT^GFP^, BrT^GFP^ and LiT^GFP^).

### Preparation of cell membranes

The collected tumour cells were lysed in a hypotonic solution containing 20 mM of Tris-HCl, 10 mM of KCl, 2 mM of MgCl_2_ and 100 mM of phenylmethanesulfonyl fluoride (Beyotime Biotechnology) for 30 min. To ensure complete membrane disruption, the lysed cells were then subjected to sonication. The lysate was centrifuged at 4 °C and 3,000*g* for 10 min to remove organelles and cellular debris. The resulting supernatant was further processed by ultracentrifugation at 50,000*g* for 1 h at 4 °C to isolate the cell membrane fragments, which were then resuspended in PBS.

### Preparation and characterization of RP@SMs

To prepare RP@SMs, 25 mg of PLGA (50:50, 7–17 kDa) and 0.5 mg of R837 (Sigma-Aldrich) were dissolved in 5 ml of dichloromethane and sonicated for 1 h to obtain a clear solution. This solution was added to 25 ml of 0.5% polyvinyl alcohol (Sigma-Aldrich) and subjected to ultrasonic emulsification. The resulting emulsion was rapidly stirred for 4 h to evaporate the dichloromethane. The solution was then centrifuged at 3,500*g* for 25 min at 4 °C to remove free R837 and large particles. The precipitate was washed three times with deionized water by centrifugation at 10,000*g* for 10 min at 4 °C, yielding the RPs.

For the preparation of membrane vesicles, plasma membranes isolated from 4T1 LuT or B16F10 cells, which transfected with STX11 or vector control, were sequentially extruded through polycarbonate membranes with pore sizes of 1 μm, 400 nm and 200 nm (Avanti Polar Lipids) using a mini-extruder (Avanti Polar Lipids), with 20 passes at each step. The resulting membrane vesicles were then co-extruded with RPs through a 200-nm membrane for 20 cycles to facilitate membrane coating. Nanoparticles derived from STX11- or vector-transfected cells were designated as RP@SMs or RP@Ms, respectively.

For characterization, 10 μl of RP@SMs was placed on a copper grid, stained with 10 μl of 2% phosphotungstic acid for 3–5 min and then air dried. The nanoparticles were imaged using a transmission electron microscope (HT7700, Hitachi) to determine their shape and size. The particle size, polydispersity index and zeta potential of the RP@SMs were measured using a Malvern Zetasizer Nano ZS90 at a fixed scattering angle of 90°. The concentration and release profile of R837 were measured at a wavelength of 265 nm using a ultraviolet–visible spectrophotometer (Shimadzu, UV-2450). The protein content of the vesicles was quantified using a BCA protein assay kit (Beyotime Biotechnology).

### Preparation of CpG-NPs

CpG-NPs were prepared as previously reported^47^. In brief, 500 µM of CpG 1826 oligonucleotides (5′-TCCATGACGTTCCTGACGTT-3′, fully phosphorothioate modified) were dissolved in 100 µl of 200-mM Tris-HCl buffer (pH 8.0) to serve as the inner aqueous phase. This solution was added to 500 µl of PLGA (50 mg ml^−1^) dissolved in dichloromethane, and emulsified by probe sonication for 1 min. The resulting water-in-oil emulsion was transferred into 5 ml of an outer aqueous phase containing 10 mM of Tris-HCl and 100 µl of dichloromethane, followed by a second round of sonication for 2 min. The double emulsion was immediately diluted into 10 ml of 10 mM Tris-HCl and stirred at 700*g* for 2.5 h. Nanoparticles were collected by centrifugation at 21,100*g* for 8 min and washed twice with 10 mM of Tris-HCl.

### In vitro uptake, activation, cytokine secretion and STX11 knockdown analysis in BMDCs

BMDCs were isolated by flushing the femurs and tibias of BALB/c mice with serum-free RPMI 1640 medium. After removing RBCs with RBC lysis buffer, the remaining cells were centrifuged to collect the cell pellet, which was then cultured in RPMI 1640 medium containing 20 ng ml^−1^ of GM-CSF (78017.2, STEMCELL Technologies) and 10 ng ml^−1^ of IL-4 (78047.2, STEMCELL Technologies) to promote DC growth and differentiation. The medium was refreshed every 2 days, and on the sixth day, adherent BMDCs were collected for further experimentation.

For *STX11* knockdown, BMDCs were transduced with lentiviral particles carrying two sgRNAs targeting sequences flanking the *STX11* coding region, cloned into the pLentiCRISPR vector. Cells were infected for 24 h and then cultured for an additional 48 h in fresh medium. Knockdown efficiency was confirmed by western blot analysis.

For uptake and activation studies, 15 μg of RP@SMs was incubated with 1 × 10^6^ BMDCs for 3, 6, 12 and 24 h. SMs were labelled with PKH67 (green), and RPs were labelled with PKH26 (red). The percentage of PKH26^+^PKH67^+^CD11c^+^ cells was analysed by flow cytometry. Additionally, 1 × 10^6^ BMDCs were incubated with PBS, RPs, SMs, RP@Ms or RP@SMs nanoparticles for 24 h. The levels of IL-6 (EK0499, SAB Signalway Antibody) and IL-12 p40 (EK14756, Sab Signalway Antibody) in the BMDC culture supernatants were quantified using ELISA kits. Flow cytometry was also used to analyse the percentage of CD80^+^CD11c^+^ or CD86^+^CD11c^+^ cells. Antibody details are provided in Supplementary Table 1.

### Antigen self-presentation to activate naive CD8^+^ T cells

T cells were isolated from the spleens of 6–8-week-old BALB/c mice using established protocols^16^. Briefly, the mice were euthanized, and their spleens were harvested, minced into small fragments and ground in 5 ml of RPMI 1640 medium using a copper mesh. The resulting cell suspension was passed through a 70-μm cell strainer to remove larger tissue debris. Following this, RBCs were lysed using RBC lysis buffer, and the remaining cells were washed three times with PBS.

To assess the ability of RP@SMs to directly activate naive CD8^+^ T cells, the isolated T cells were co-cultured with PBS, RPs, SMs, RP@Ms or RP@SMs nanoparticles in RPMI 1640 medium. After 24 h of incubation, the T cells were collected and sequentially incubated with FITC-CD3 and PerCP-CD8 antibodies for 15 min, followed by staining with Fixable Viability Dye eFluor 450 (65-0863-14, eBioscience) in the dark at 4 °C for 15 min. The cells were then incubated with PE/Cy7-IFNγ and APC-TNFα antibodies for an additional 15 min. After washing the samples with PBS, flow cytometry was performed using a CytoFLEX S flow cytometer (Beckman).

The proliferation of CD8^+^ T cells was also assessed. Initially, T cells were incubated with carboxyfluorescein succinimidyl ester (C34554, Life Technologies) at 37 °C in the dark for 20 min, followed by three washes with PBS. The labelled T cells were then co-cultured with PBS, RPs, SMs, RP@Ms or RP@SMs nanoparticles in RPMI 1640 medium for 24 h. Following this incubation, the cells were stained with APC-CD3 and PerCP-CD8 antibodies for 15 min, washed with PBS and analysed by flow cytometry. Antibody details are provided in Supplementary Table 1.

### In vitro cytotoxicity of activated T cells against tumour cells

To assess the cytotoxic capacity of activated T cells, T cells were either directly incubated with various nanoparticle formulations or indirectly primed via co-culture with BMDCs pretreated with the respective nanoparticles (DC-to-T ratio of 1:10) for 24 h. The activated T cells were then co-cultured with 4T1 LuT tumour cells at an effector-to-target ratio of 10:1 for 24 h. Tumour cell lysis was quantified using a lactate dehydrogenase release assay (Beyotime Biotechnology) following the manufacturer’s protocol, serving as a measure of T cell-mediated cytotoxicity.

### In vivo imaging and biosafety of RP@SMs

For LN drainage studies, BALB/c mice were subcutaneously injected with DiD-labelled RP@Ms or RP@SMs in the right dorsal side. Imaging was conducted 12 h post-injection using the Maestro in vivo imaging system (PerkinElmer). To evaluate the biosafety of RP@SMs, BALB/c mice were administered PBS or RP@SMs at a dose of 5 mg kg^−1^. The mice were euthanized 24 h later, and blood samples were collected for biochemical and haematological analysis. Major organs were harvested and subjected to histological examination via H&E staining.

### Animal models and treatments

Orthotopic breast tumour model: to compare tumourigenicity, BALB/c mice received orthotopic injections of 5 × 10^5^ 4T1 LuT or 4T1 Parental cells into the left mammary fat pad. Tumour growth was monitored every other day, and mice were euthanized when tumours reached 1,000 mm^3^.

Postoperative metastatic breast cancer model: to mimic clinical postoperative recurrence, 5 × 10^5^ 4T1 LuT^Luc^ cells were inoculated into the left mammary fat pad. On day 6, when tumours reached ~100 mm^3^, mice were randomized into five groups (*n* = 10) and treated subcutaneously with PBS, RPs, SMs, RP@Ms or RP@SMs (100 μl) on days 6, 9 and 13. On day 20, mice were euthanized and the lungs were collected for the histological assessment of metastatic burden by H&E staining. Tumour volume (*V*) was calculated as *V* = width^2 ^× length/2.

T cell depletion experiments: to assess the role of T lymphocyte subsets, mice were injected intraperitoneally with anti-CD4 (250 μg, BP0003, BioXCell), anti-CD8α (250 μg, BP0061, BioXCell) or isotype control (250 μg, BP0090, BioXCell) antibodies on days 5, 8, 11, 14 and 17 post-inoculation. RP@SMs (5 mg kg^−1^ in 100 μl PBS) were administered subcutaneously on days 6, 9, 13 and 20. Tumour growth was recorded every other day, and mice were euthanized at 1,000 mm^3^.

DC depletion: CD11c-DTR mice (Aniphe Biolaboratory) were used as previously described^48^. For transient depletion, DTx (20 ng g^−1^, D0564, Sigma-Aldrich) was administered intraperitoneally 1 day before RP@SMs vaccination, and spleens and dLNs were harvested on day 1 post-vaccination for flow cytometry. For sustained depletion, DTx was administered every other day starting 1 day before RP@SMs injection until euthanasia. Tumour growth was monitored every other day, and mice were euthanized when tumour volumes reached 1,000 mm^3^ in accordance with ethical guidelines.

Postoperative tumour recurrence model: for the recurrence model, mice were inoculated with tumour cells and randomized to five groups (*n* = 10) when tumours reached ~60 mm^3^ on day 5. Incomplete resection (~10% tumour left in situ) was followed by subcutaneous PBS, RPs, SMs, RP@Ms or RP@SMs (100 μl) on days 6, 9 and 13. Tumour volumes were measured every other day until day 22.

Combination therapy with PD-L1 blockade: to assess synergy, mice with ~60-mm^3^ tumours on day 5 underwent 90% resection and were randomized to four groups (*n* = 10). RP@SMs (5 mg kg^−1^) were administered subcutaneously on days 6, 9, 13 and 20; anti-PD-L1 antibody (BP0101, BioXCell; 100 μg per mouse) was given intravenously on days 8, 11 and 14. Tumour growth was monitored every other day; mice were euthanized when tumours reached 1,000 mm^3^.

Long-term immune memory: mice surviving RP@SMs treatment were rechallenged with 5 × 10^5^ 4T1 LuT cells on day 80; age-matched naive mice served as controls. Tumour growth was monitored until humane endpoints.

Personalized RP@SMs therapy in breast cancer: for personalized vaccination, mice with ~60-mm^3^ tumours on day 5 underwent incomplete resection (~10% tumour left). Resected tumours were digested with collagenase IV, hyaluronidase and DNase I to obtain single-cell suspensions. On day 11, donor tumour cells were transfected with *STX11*-expressing plasmids (SM and RP@SMs groups) or control vectors (RP@M group). Cell membrane vesicles were harvested on day 13, co-extruded with RPs and administered subcutaneously (days 13, 16 and 20). Tumour growth was monitored every other day until day 22.

Personalized RP@SMs therapy in melanoma: B16F10 cells (5 × 10^5^) were subcutaneously inoculated into the right flank of C57BL/6 male mice. On day 5 (tumour volume, ~70 mm^3^), incomplete resection was performed, leaving approximately 10% of the tumour. On day 8, tumour cells were transfected with *STX11* or control plasmids, and personalized nanovaccines were prepared on day 10. Mice received subcutaneous injections in the left abdomen on days 10, 13 and 17, with PBS as the control. Tumour progression was monitored every other day from day 2 after vaccination until day 18.

Personalized RP@DM therapy: RP@DM treatment followed the same procedure as RP@SMs therapy, except tumour cells were treated with DPT on day 11. Artificial nanovaccines were prepared by day 12 and administered subcutaneously on days 12, 15 and 19. Tumour growth was monitored every other day until day 22.

### In vivo immune analysis

Following treatment, TDLNs, spleens and tumour tissues were harvested from 4T1-LuT^Luc^-tumour-bearing mice. The tumour tissues were finely minced and digested with type IV collagenase, hyaluronidase and DNase I (Sigma-Aldrich) at 37 °C for 1 h. The resulting tumour homogenates were centrifuged at 350*g* for 5 min, and RBCs were lysed using RBC lysis buffer. The remaining cells were resuspended in PBS and passed through a 70-μm cell strainer to obtain a single-cell suspension. Spleens and TDLNs were mechanically disrupted, followed by RBC lysis and filtration through a 70-μm cell strainer to prepare single-cell suspensions. All cell suspensions were maintained in PBS containing 2% fetal bovine serum.

For cell surface marker analysis, cells were first blocked with CD16/32 antibody for 15 min to prevent non-specific binding. The cells were then incubated at room temperature for 15 min with various fluorescent antibodies, including APC-CD11c, FITC-CD80, PE/Cyanine7-CD86, FITC-CD3, PerCP/Cyanine5.5-CD8, APC-CD44, PE-CD62L, eFluor 450-CD45, PE-PD-1 and APC-PD-L1, following the manufacturer’s instructions. Subsequently, cells were stained with SYTOX AADvanced Dead Cell Stain (S10349, Thermo Fisher) at 4 °C for 15 min. Data acquisition was performed using a CytoFLEX S flow cytometer (Beckman), and the results were analysed using FlowJo software (v10.9, BD Biosciences). Detailed information on the antibodies used is provided in Supplementary Table 1.

### Sodium dodecyl sulfate–polyacrylamide gel electrophoresis and western blot analysis

Cellular and membrane proteins were extracted using a protein extraction kit (Beyotime Biotechnology) and analysed using sodium dodecyl sulfate–polyacrylamide gel electrophoresis according to standard protocols. Western blotting was performed following established procedures^27^. Protein complexes were visualized using the ChemiDOC system (Bio-Rad Laboratories) and quantified with Image Lab software (v. 4.0; Bio-Rad). Specific information about the antibodies used is detailed in Supplementary Table 2.

### Plasmid transfection

Human and mouse STX11 sequences (NM_003764.4 for humans and NM_001163590.1 for mice) were cloned into the pcDNA3.1(+) vector (GENERAL BIOL). Human shSTX11, mouse shSTX11 and non-targeting shRNAs were synthesized by GENERAL BIOL. During transfection, Lipofectamine 3000 (Invitrogen) was used to introduce plasmids or shRNAs into tumour cells according to the manufacturer’s instructions. After 48 h, the transfected tumour cells were analysed by western blot and RT-qPCR. The shRNA sequences are listed in Supplementary Table 3.

### RT-qPCR

RT-qPCR was conducted as previously described^49^. Total RNA was extracted using an RNA extraction kit (ESscience) following the manufacturer’s instructions. The extracted RNA was reverse transcribed into cDNA using a reverse transcription kit (Vazyme). RT-qPCR was performed using SYBR Green Master Mix (Vazyme) and a LightCycler 480 detector (Roche). Primer sequences are detailed in Supplementary Table 3.

### Statistics and reproducibility

Statistical analysis was performed using GraphPad Prism (v. 8.0.2) software. All experiments were independently repeated at least three times, and data are presented as mean ± standard deviation (mean ± s.d.). Statistical significance was determined using a one-way analysis of variance (ANOVA) with Tukey’s multiple comparisons test, an unpaired two-sided *t*-test or a log-rank (Mantel–Cox) test. A *P* value of less than 0.05 was considered statistically significant.

### Reporting summary

Further information on research design is available in the Nature Portfolio Reporting Summary linked to this article.

## Online content

Any methods, additional references, Nature Portfolio reporting summaries, source data, extended data, supplementary information, acknowledgements, peer review information; details of author contributions and competing interests; and statements of data and code availability are available at 10.1038/s41565-025-02113-w.

## Supplementary information

Supplementary Information Supplementary Figs. 1–28 and Supplementary Tables 1–3.

Reporting Summary

Supplementary Data 1 Source data for Supplementary Figures.

Supplementary Data 2 STR profiling analysis of cell lines.

## Source data

Source Data Fig. 1 Statistical source data.

Source Data Fig. 2 Statistical source data.

Source Data Fig. 3 Unprocessed western blots.

Source Data Fig. 4 Statistical source data.

Source Data Fig. 5 Statistical source data.

Source Data Fig. 6 Statistical source data.

Source Data Extended Data Fig. 1 Statistical source data.

Source Data Extended Data Fig. 2 Unprocessed western blots.

Source Data Extended Data Fig. 3 Statistical source data.

## Data Availability

The main data supporting the results in this study are available within the Article and its Supplementary Information. Transcriptomic analyses and clinical information data were obtained from the TCGA and GTEx databases (http://sangerbox.com/tool.html) and TIMER2.0 (http://timer.cistrome.org/). The raw single-cell RNA-sequencing data have been deposited in the Gene Expression Omnibus (GEO) under accession number GSE199219. Source data are provided with this paper.
