# Supplementary material for: Biomimetic vesicles engineered from modified tumour cells act as personalized vaccines for post-surgical cancer immunotherapy
Source: Nat Nanotechnol. 2026 Jan 29;21(3):443–54. doi: 10.1038/s41565-025-02113-w (PMC13017505; doi:10.1038/s41565-025-02113-w)
Supplement: Supplementary file 1 — Supplementary Figs. 1–28 and Supplementary Tables 1–3. [file 41565_2025_2113_MOESM1_ESM.pdf]

nature nanotechnology

Supplementary information https://doi.org/10.1038/s41565-025-02113-w

# Biomimetic vesicles engineered from modified tumour cells act as personalized vaccines for post-surgical cancer immunotherapy

In the format provided by the authors and unedited

# Table of contents

**Fig. S1** *STX11* expression negatively correlates with disease progression in cancer patients.

**Fig. S2** STX11 regulates antigen presentation and maturation of BMDCs.

**Fig. S3** Negative correlation between *STX11* expression and tumor size in BRCA.

**Fig. S4** Positive correlation between *STX11* expression and *MHC*, *CD80/86*, and tumor immune infiltration in BRCA.

**Fig. S5** Overexpression of STX11 promotes MHC I expression in postoperative lung-metastatic TNBC cells.

**Fig. S6** Overexpression of STX11 promotes surface CD80/CD86 expression in TNBC cells.

**Fig. S7** Knockdown of STX11 suppresses surface MHC I expression in TNBC cells.

**Fig. S8** Characterization of RP@SMs.

**Fig. S9** LN targeting by RP@SMs.

**Fig. S10** Biocompatibility of RP@SMs.

**Fig. S11** DC uptake by RP@SMs.

**Fig. S12** Flow cytometry analysis of $CD8^+$ T cells from mouse spleens.

**Fig. S13** *In vivo* tumor progression and survival analysis of 4T1 LuT and 4T1 Parental tumors.

**Fig. S14** RP@SMs inhibit tumor lung metastasis.

**Fig. S15** Immune response induced by RP@SMs in TDLNs.

**Fig. S16** Immune response induced by RP@SMs in the spleen.

**Fig. S17** Immune response induced by RP@SMs in tumors.

**Fig. S18** DTx-mediated depletion of $CD11c^+$ DCs in CD11c-DTR mice.

**Fig. S19** Tumor growth kinetics of 4T1 LuT cells in CD11c-DTR mice.

**Fig. S20** RP@SMs inhibit postoperative lung metastasis in TNBC.

**Fig. S21** Effects of RP@SMs nanovaccine on PD-L1 expression on tumor cells and PD-1 expression on $CD8^+$ tumor-infiltrating lymphocytes.

**Fig. S22** Effect of RP@SMs on mouse body weight.

**Fig. S23** Lack of therapeutic efficacy of heterologous B16F10-derived RP@SMs in 4T1 LuT tumor-bearing mice.

**Fig. S24** Effects of DPT on cell viability and STX11 protein expression in postoperative lung-metastatic TNBC cells.

**Fig. S25** DPT enhances surface MHC I expression in postoperative lung-metastatic TNBC cells.

**Fig. S26** DPT enhances surface expression of CD80 and CD86 in postoperative lung-metastatic TNBC cells.

**Fig. S27** DPT-based RP@DMs personalized autologous vaccine for the treatment of postoperative metastatic TNBC.

**Fig. S28** Unprocessed images of all gels and blots used in the Supplementary Figures.

**Supplementary Table 1** Flow cytometry antibodies used in this study.

**Supplementary Table 2** Western blot antibodies used in this study.

**Supplementary Table 3** Sequences of RT-qPCR, shRNA and sgRNA primers.

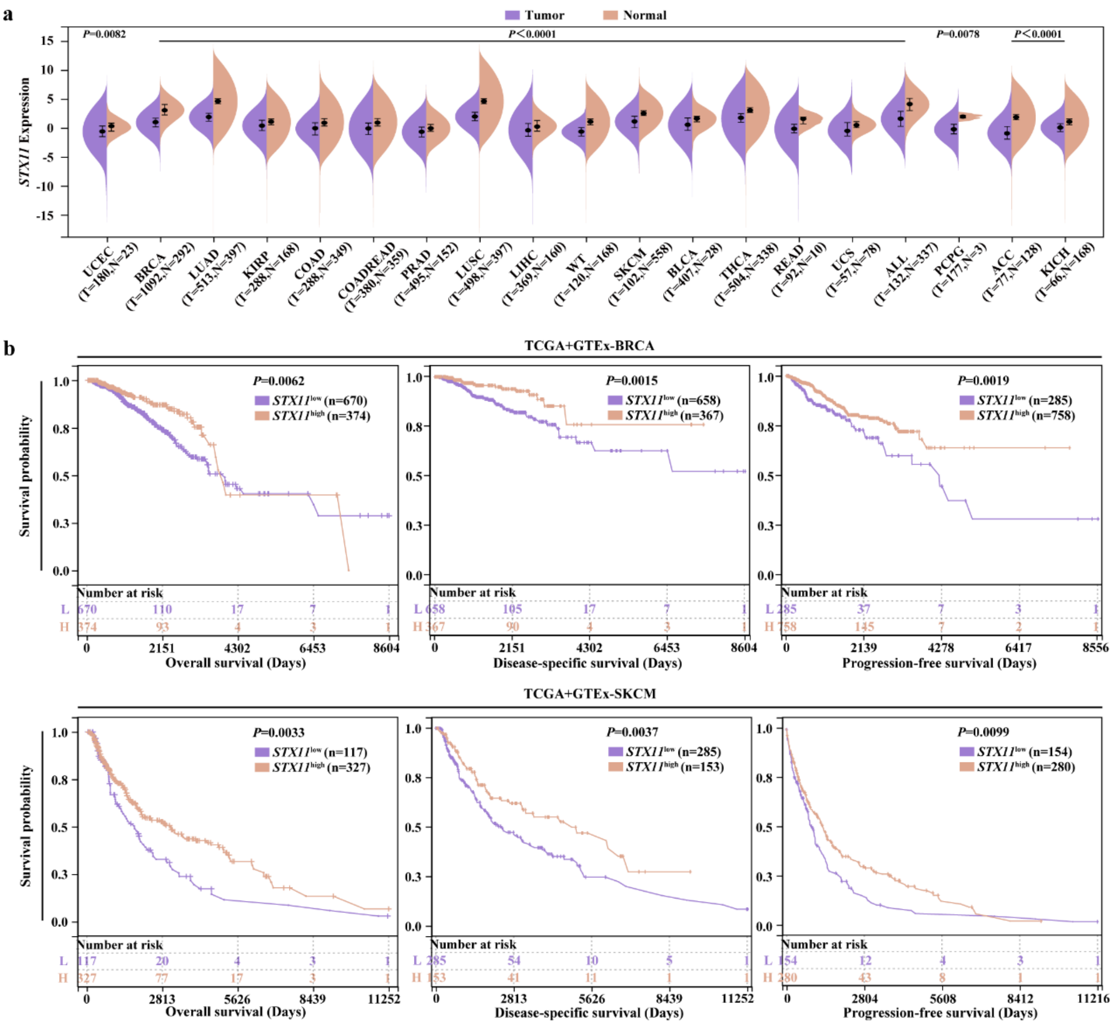


**Fig. S1 *STX11* expression negatively correlates with disease progression in cancer patients. a**, *STX11* gene expression in normal and tumor samples from patients with uterine corpus endometrial carcinoma (UCEC), breast cancer (BRCA), lung adenocarcinoma (LUAD), kidney papillary cell carcinoma (KIRP), colon adenocarcinoma (COAD), colorectal adenocarcinoma (COADREAD), prostate cancer (PRAD), lung squamous carcinoma (LUSC), hepatocellular carcinoma (LIHC), Wilms tumor (WT), melanoma (SKCM), bladder urothelial carcinoma (BLCA), thyroid cancer (THCA), rectal adenocarcinoma (READ), uterine sarcoma (UCS), leukemia (ALL), pheochromocytoma and paraganglioma (PCPG), adrenocortical carcinoma (ACC), and kidney chromophobe carcinoma (KICH). **b**, Kaplan-Meier survival analysis (overall survival, disease-specific survival, and progression-free survival) of BRCA and SKCM patients with high versus low *STX11* expression.

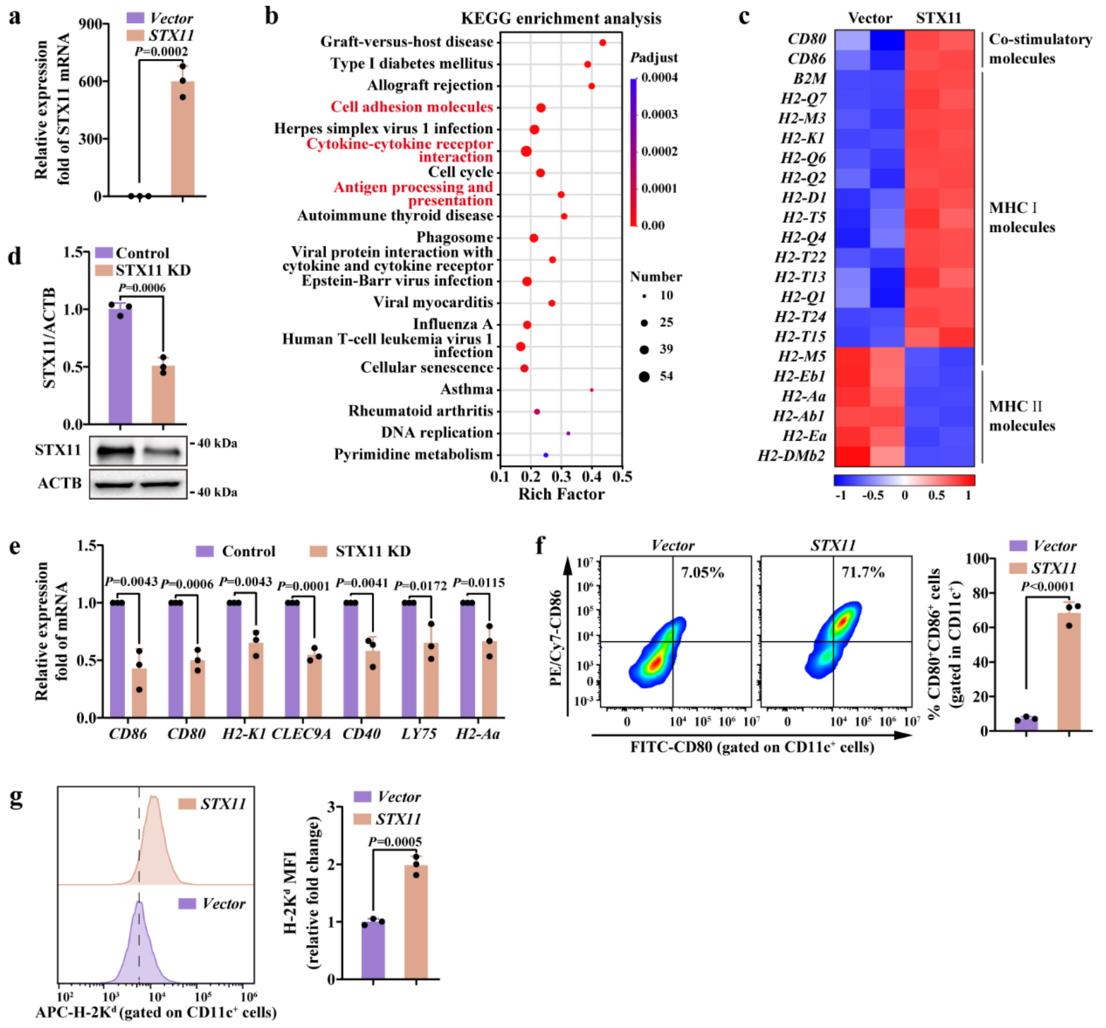


**Fig. S2 STX11 regulates antigen presentation and maturation of BMDCs. a**, Quantification of *STX11* mRNA expression in BMDCs transfected with *STX11* or *Vector* ($n = 3$ independent biological replicates). **b**,**c**, Transcriptomic profiling of BMDCs transfected with *STX11* or *Vector* ($n = 2$ independent biological replicates). **b,** Kyoto Encyclopedia of Genes and Genomes (KEGG) pathway enrichment analysis showing the top 20 significantly altered pathways. **c**, Heatmap of differentially expressed genes related to MHC I/MHC II molecules and co-stimulatory molecules. Genes with $P$ adjust < 0.05 and $|\log_2$ fold change$|$ > 1 are highlighted. **d**,**e**, Quantification of (**d**) STX11 protein and (**e**) selected immune-related genes in BMDCs transfected with *STX11-sgRNA* (*sgRNA1* + *sgRNA2*) or control *vector* ($n = 3$ independent biological replicates). **f**,**g**, Flow cytometry analysis of (**f**) MHC I and (**g**) CD80/CD86 surface expression on CD11c$^+$ BMDCs after transfection with *STX11* or *Vector* ($n = 3$ independent biological replicates). Data are presented as mean ± s.d.

Statistical significance was determined using an unpaired two-sided t-test with a confidence interval of 95%.

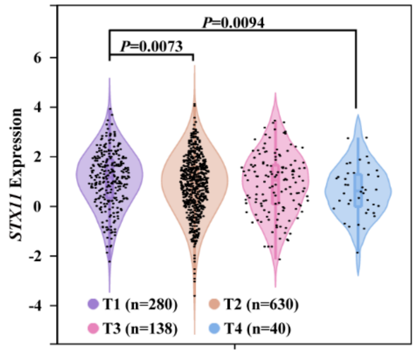


**Fig. S3 Negative correlation between *STX11* expression and tumor size in BRCA.** Correlation between tumor size and *STX11* gene expression in BRCA patients.

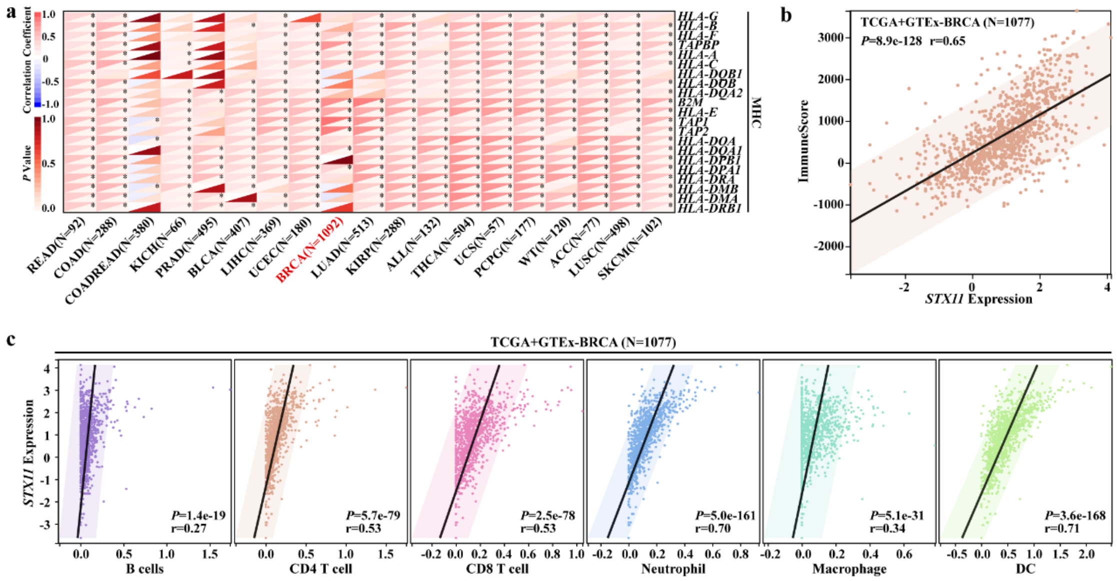


**Fig. S4 Positive correlation between *STX11* expression and *MHC*, *CD80/86*, and tumor immune infiltration in BRCA. a**, Pearson correlation between *STX11* and MHC molecules across 19 cancer types. **b**, Correlation between *STX11* expression and immune score in BRCA patients. **c**, Correlation between *STX11* expression and immune cell infiltration in BRCA patients.

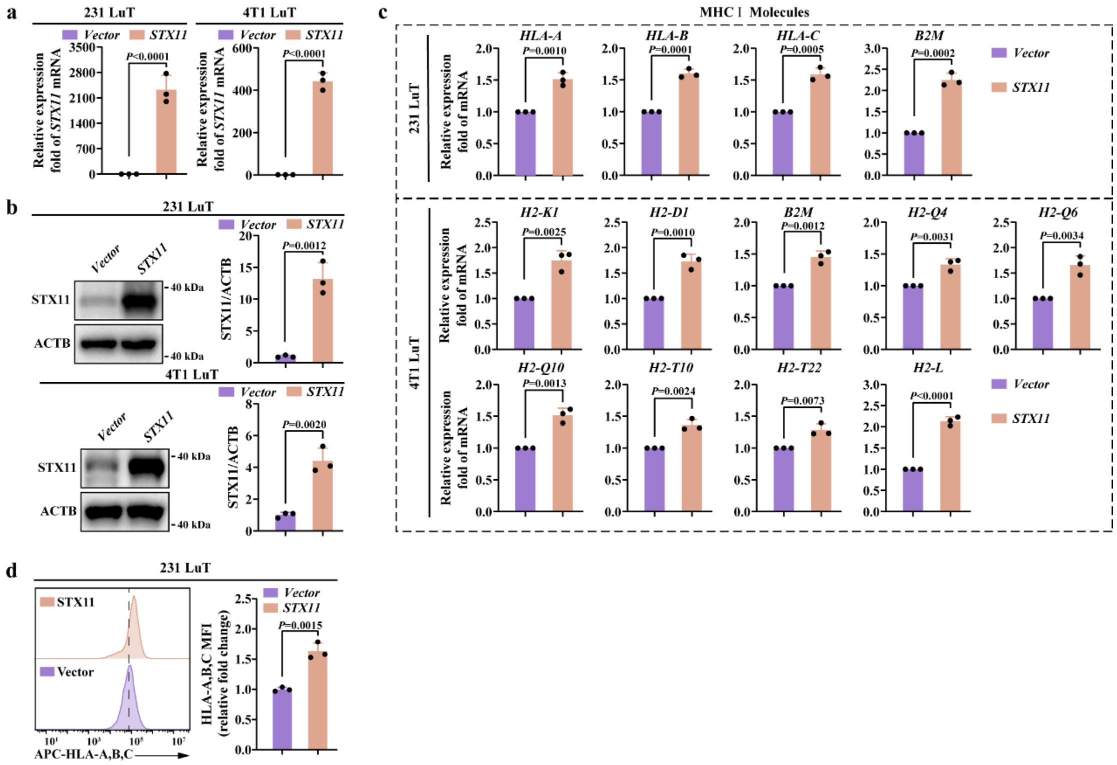


**Fig. S5 Overexpression of STX11 promotes MHC I expression in postoperative lung-metastatic TNBC cells. a**,**b**, Quantification of STX11 gene (**a**) and protein (**b**) expression in lung-metastatic TNBC cells transfected with *STX11* or *Vector* ($n = 3$ independent biological replicates). **c**, RT-qPCR analysis of *MHC I* gene expression in *STX11*- or *Vector*-transfected lung-metastatic TNBC cells ($n = 3$ independent biological replicates). **d**, Flow cytometry analysis of surface MHC I levels in 231 LuT cells transfected with *STX11* or *Vector* ($n = 3$ independent biological replicates). Data are presented as mean ± s.d. Statistical significance was determined using an unpaired two-sided t-test with a confidence interval of 95%.

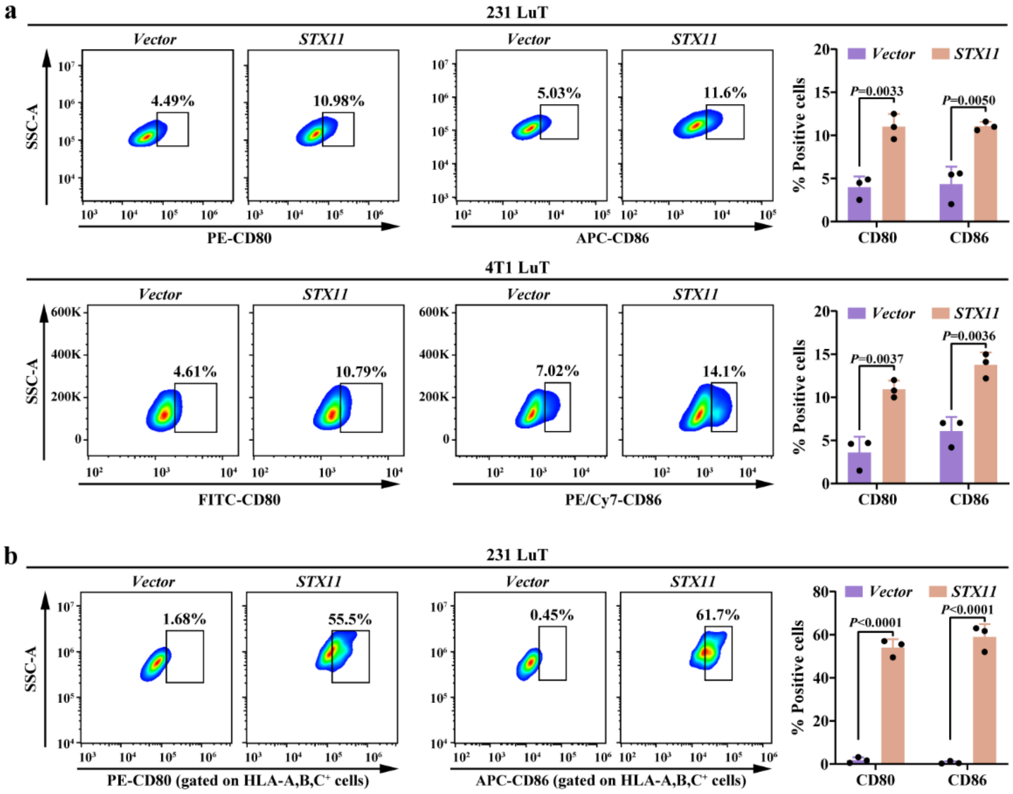


**Fig. S6 Overexpression of STX11 promotes surface CD80/CD86 expression in TNBC cells. a**, Flow cytometry analysis of surface CD80/CD86 levels in postoperative lung-metastatic TNBC cells transfected with *STX11* or *Vector* ($n = 3$ independent biological replicates). **b**, Flow cytometry analysis of the percentages of $CD80^{+}MHC\ I^{+}$ and $CD86^{+}MHC\ I^{+}$ cell populations in 231 LuT cells transfected with *STX11* or *Vector* ($n = 3$ independent biological replicates). Data are presented as mean ± s.d. Statistical significance was determined using an unpaired two-sided t-test with a confidence interval of 95%.

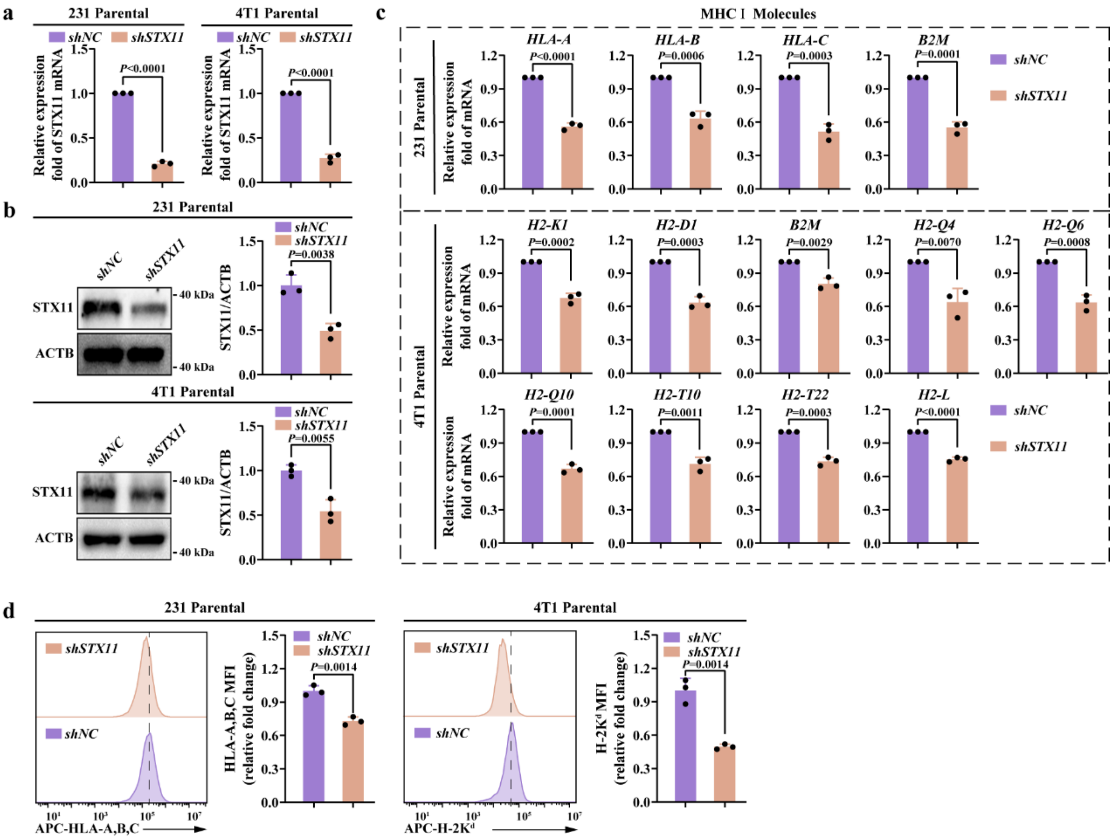


**Fig. S7 Knockdown of STX11 suppresses surface MHC I expression in TNBC cells.** **a**,**b**, Quantification of STX11 gene (**a**) and protein (**b**) expression in parental TNBC cells transfected with *shSTX11* or *shNC* ($n = 3$ independent biological replicates). **c**, RT-qPCR analysis of *MHC I* gene expression in TNBC parental cells transfected with *shSTX11* or *shNC* ($n = 3$ independent biological replicates). **d**, Flow cytometry analysis of surface MHC I levels in TNBC parental cells transfected with *shSTX11* or *shNC* ($n = 3$ independent biological replicates). Data are presented as mean ± s.d. Statistical significance was determined using an unpaired two-sided t-test with a confidence interval of 95%.

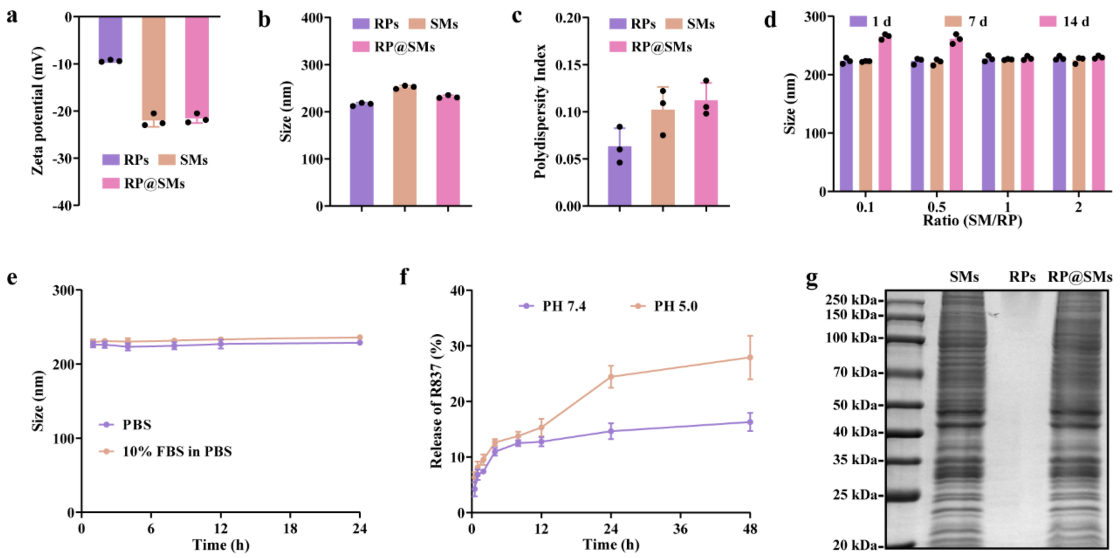


**Fig. S8 Characterization of RP@SMs. a-c**, Zeta potential (**a**), particle size (**b**), and polydispersity index (PDI) (**c**) of RPs, SMs, and RP@SMs nanoparticles (*n* = 3 independent biological replicates). **d**, Diameter of RP@SMs at different SM-to-RP ratios (*n* = 3 independent biological replicates). **e**, Diameter of RP@SMs in PBS and PBS containing 10 % FBS at 37 °C (*n* = 3 independent biological replicates). **f**, R837 release profile from RP@SMs (*n* = 3 independent biological replicates). **g**, SDS-PAGE analysis of SMs, RPs, and RP@SMs.

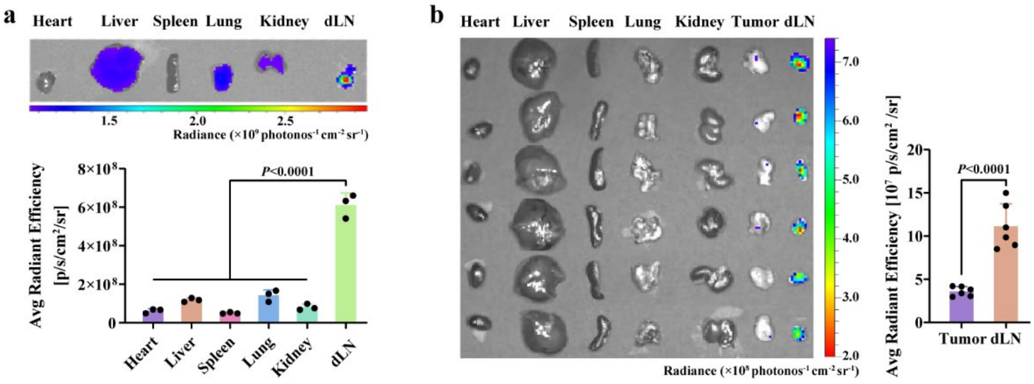


**Fig. S9 LN targeting by RP@SMs. a**,**b**, IVIS imaging was performed 12 hours after subcutaneous injection of DiD-labeled RP@SMs into the right dorsal flank of mice. Fluorescence signals were detected in major organs, and the mean fluorescence intensities were quantified for (**a**) each organ (*n* = 3 mice per group) and (**b**) tumors and dLNs (*n* = 6 mice per group). Data are presented as mean ± s.d. Statistical significance

was determined using a one-way ANOVA with Tukey’s multiple-comparisons test or an unpaired two-sided t-test with a confidence interval of 95%.

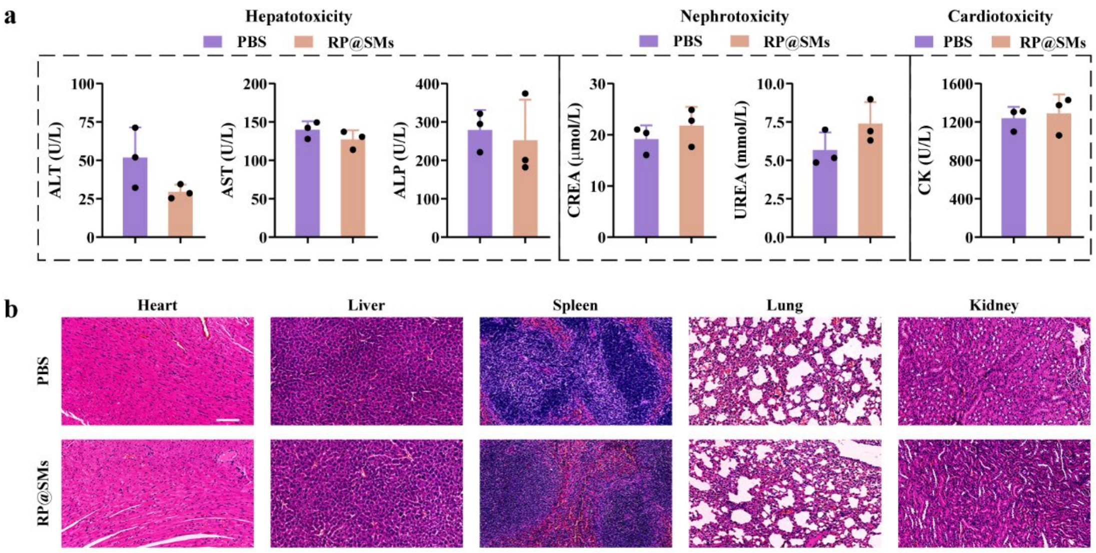


**Fig. S10 Biocompatibility of RP@SMs. a**,**b**, (**a**) Plasma biochemical analysis of alanine aminotransferase (ALT), aspartate aminotransferase (AST), alkaline phosphatase (ALP), creatinine (CREA), urea (UREA), and creatine kinase (CK) levels and (**b**) H&E-stained histopathological analysis of major organs from mice injected with PBS or RP@SMs ($n = 3$ mice per group). Scale bars, 100 μm.

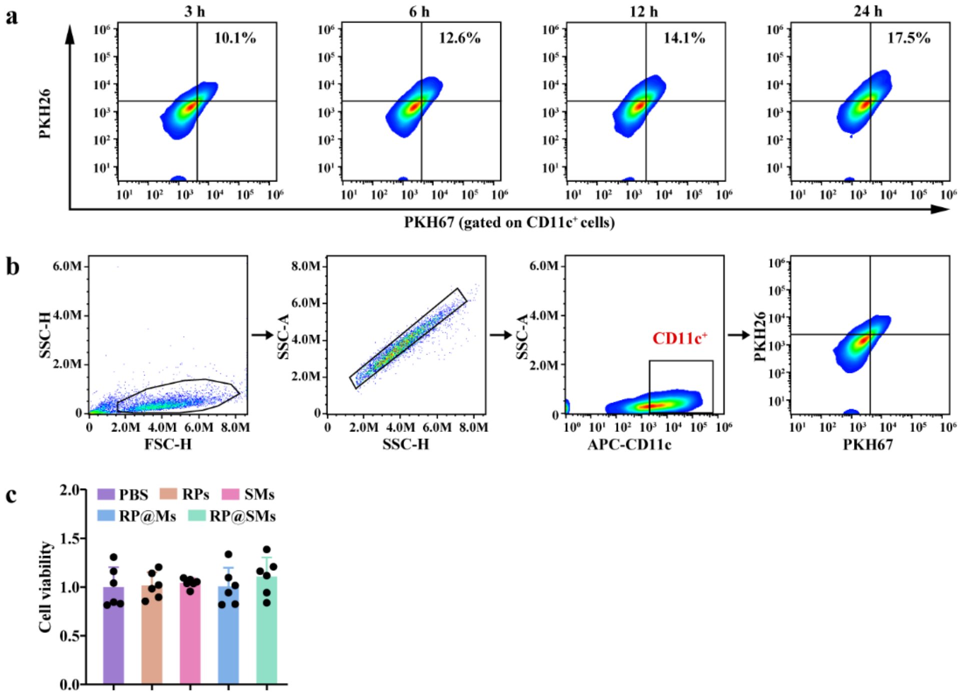

**Fig. S11 DC uptake by RP@SMs a**, Representative flow cytometry images showing BMDC uptake of RP@SMs (PKH26-labeled RPs, PKH67-labeled SMs) over time. **b**, Gating strategy used for the analysis in panel **a**. **c**, Cell viability assays for BMDCs after different nanoparticles (*n* = 6 independent biological replicates).

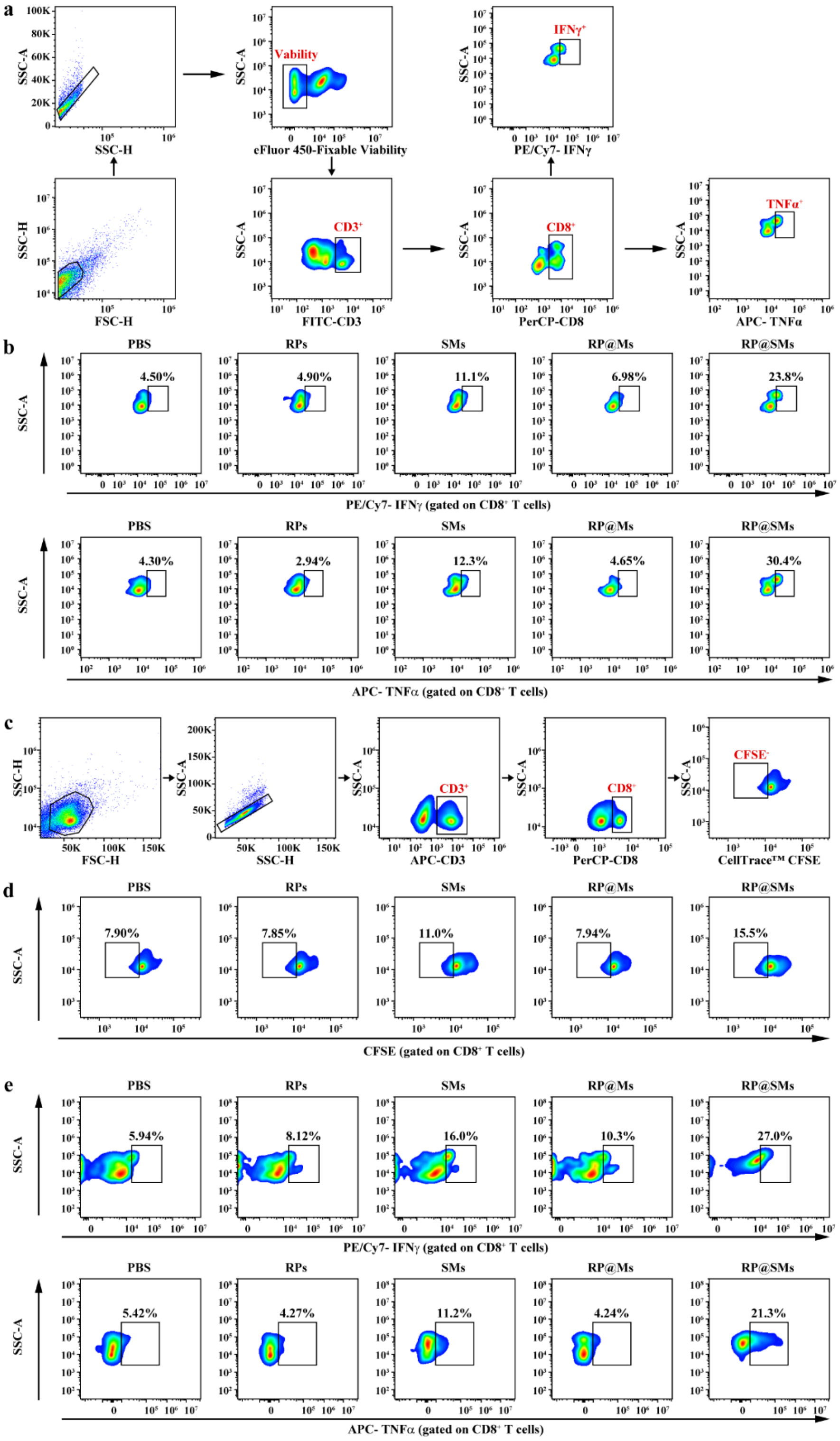

**Fig. S12 Flow cytometry analysis of CD8$^+$ T cells from mouse spleens. a**,**c**, Gating strategy used for the analysis shown in panels **b** and **d**. **b**,**d**, Flow cytometry analysis showing the percentage of (**b**) IFNγ$^+$CD8$^+$ and TNFα$^+$CD8$^+$ T cells, and (**d**) proliferating CD8$^+$ T cells from mouse spleens. **e**, Flow cytometry analysis of the percentages of IFNγ$^+$CD8$^+$ and TNFα$^+$CD8$^+$ T cells in the DC-to-T co-culture system, following 24-hour incubation of BMDCs stimulated with different nanoparticles and splenocytes at a 1:10 ratio.

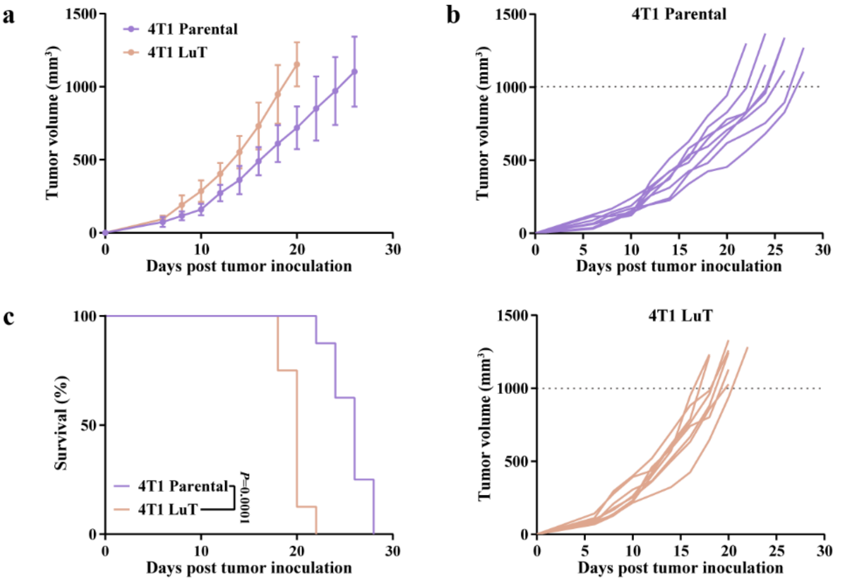


**Fig. S13 *In vivo* tumor progression and survival analysis of 4T1 LuT and 4T1 Parental tumors. a**, Average tumor growth curves of mice bearing 4T1 LuT or 4T1 Parental tumors ($n = 8$ mice per group). **b**, Individual tumor growth trajectories for each mouse ($n = 8$ mice per group). **c**, Kaplan-Meier survival curves of tumor-bearing mice ($n = 8$ mice per group). Data are presented as mean ± s.d. Statistical significance was determined using a log-rank (Mantel-Cox) test with a confidence interval of 95%.

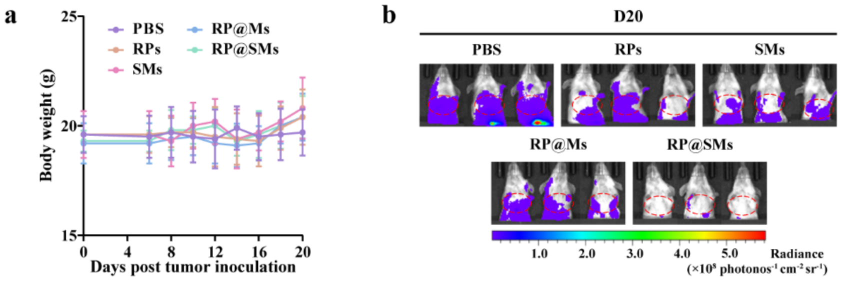

**Fig. S14 RP@SMs inhibit tumor lung metastasis. a**, Body weight changes in 4T1 LuT$^{Luc}$ tumor-bearing mice after different treatments ($n = 10$ mice per group). **b**, Bioluminescence images of lungs from 4T1 LuT$^{Luc}$ tumor-bearing mice at day 20 after various treatments with a confidence interval of 95%.

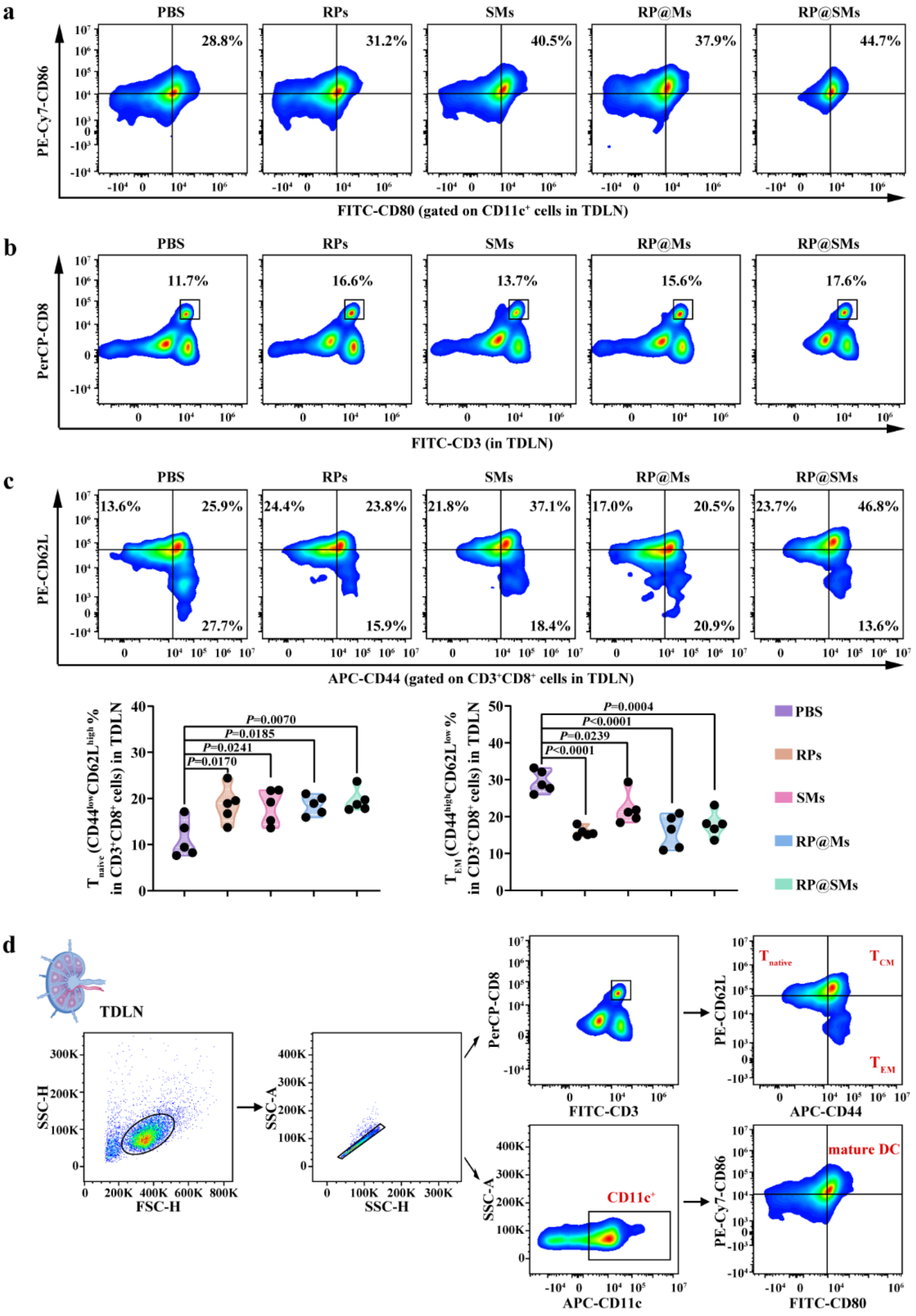


**Fig. S15 Immune response induced by RP@SMs in TDLNs. a**, Representative flow cytometry images of mature DCs (CD11c$^+$CD80$^+$CD86$^+$ cells) in TDLNs. **b**,

Representative flow cytometry images of CTLs ($CD3^+CD8^+$ cells) in TDLNs. **c**, Representative flow cytometry images of naive $CD8^+$ T ($T_{naive}$) cells and effector memory $CD8^+$ T ($T_{EM}$) cells in TDLNs, with quantification of $T_{naive}$ and $T_{EM}$ cells ($n = 5$ mice per group). **d**, Gating strategy for the experiments shown in panels **a**-**c**. Data are presented as mean ± s.d. Statistical significance was determined using a one-way ANOVA with Tukey's multiple-comparisons test with a confidence interval of 95%. Panel **d** created with BioRender.com.

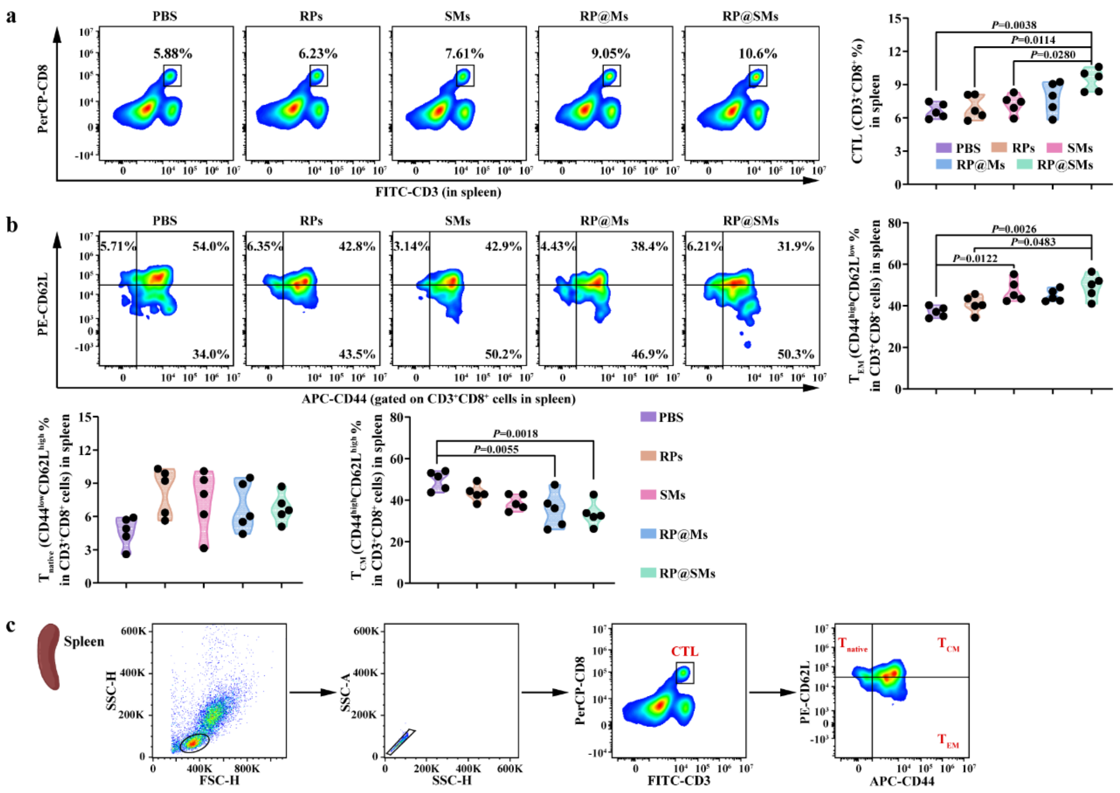


**Fig. S16 Immune response induced by RP@SMs in the spleen.** **a**, Representative flow cytometry images and quantification of CTLs in the spleen ($n = 5$ mice per group). **b**, Representative flow cytometry images and quantification of $T_{naive}$, $T_{CM}$, and $T_{EM}$ cells in the spleen ($n = 5$ mice per group). **c**, Gating strategy used for the experiments shown in panels **a** and **b**. Data are presented as mean ± s.d. Statistical significance was determined using a one-way ANOVA with Tukey's multiple-comparisons test with a confidence interval of 95%. Panel **c** created with BioRender.com.

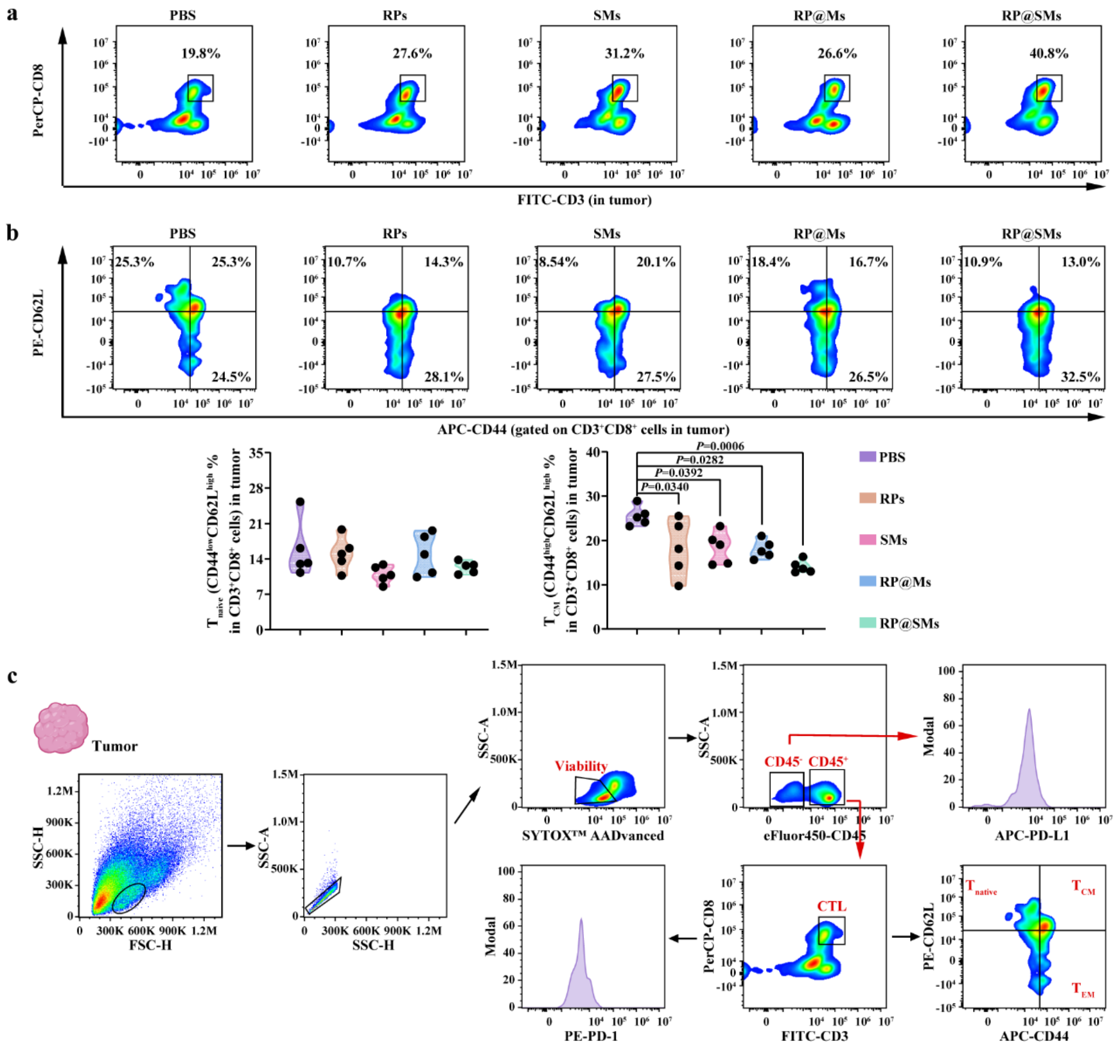


**Fig. S17 Immune response induced by RP@SMs in tumors. a**, Representative flow cytometry images of CTLs in tumor tissues. **b**, Representative flow cytometry images and quantification of $T_{naive}$ and $T_{CM}$ cells in tumor tissues ($n = 5$ mice per group). **c**, Gating strategy for experiments in Fig. S17a-b and S21a-b. Data are presented as mean ± s.d. Statistical significance was determined using a one-way ANOVA with Tukey's multiple-comparisons test with a confidence interval of 95%. Panel **c** created with BioRender.com.

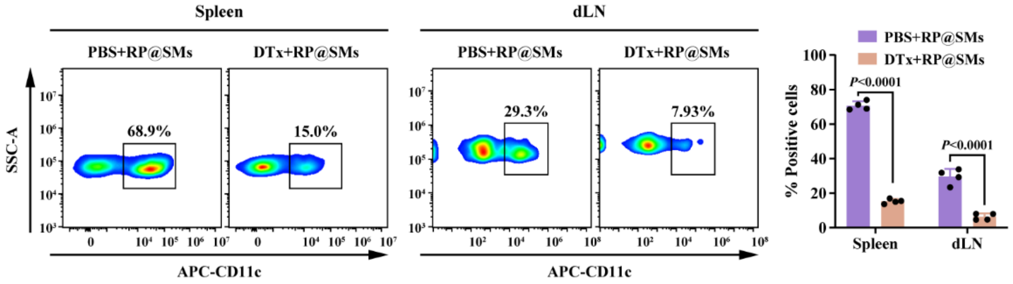


**Fig. S18 DTx-mediated depletion of CD11c$^+$ DCs in CD11c-DTR mice.** Representative flow cytometry plots and quantitative analysis of CD11c$^+$ cells in the spleens and dLNs of CD11c-DTR mice treated with PBS or DTx and subsequently immunized with RP@SMs ($n = 4$ mice per group). Data are presented as mean ± s.d. Statistical significance was determined using an unpaired two-sided t-test with a confidence interval of 95%.

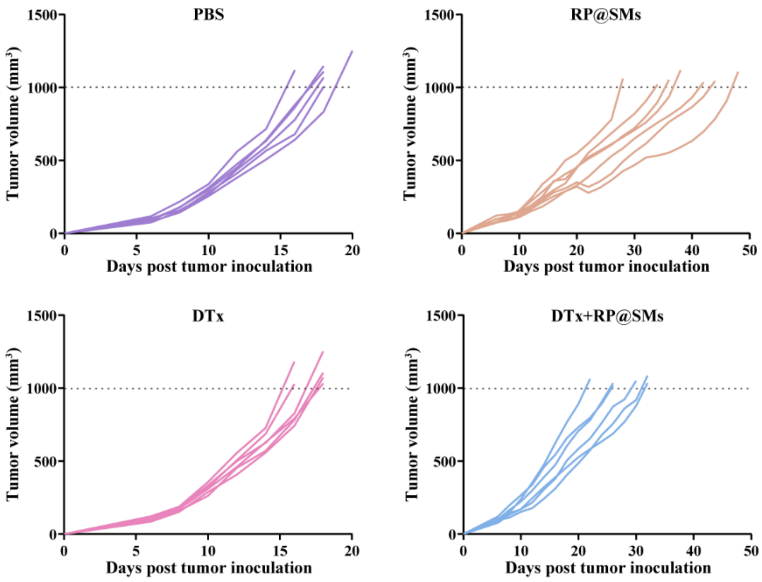


**Fig. S19 Tumor growth kinetics of 4T1 LuT cells in CD11c-DTR mice.** Individual tumor growth curves in mice treated with RP@SMs, with or without DTx-mediated transient DC depletion ($n = 6$-7 mice per group).

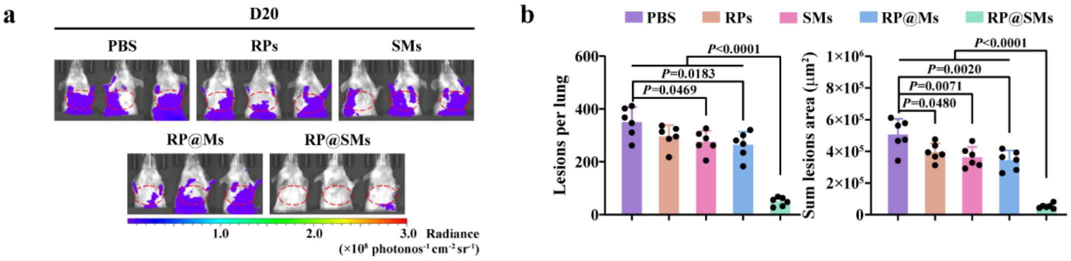

**Fig. S20 RP@SMs inhibit postoperative lung metastasis in TNBC. a,** Bioluminescence images of lungs from 4T1 $LuT^{Luc}$ tumor-bearing mice at day 20 after various treatments. **b**, Quantification of lung metastases across treatment groups ($n = 6$ mice per group). Data are presented as mean ± s.d. Statistical significance was determined using a one-way ANOVA with Tukey's multiple-comparisons test with a confidence interval of 95%.

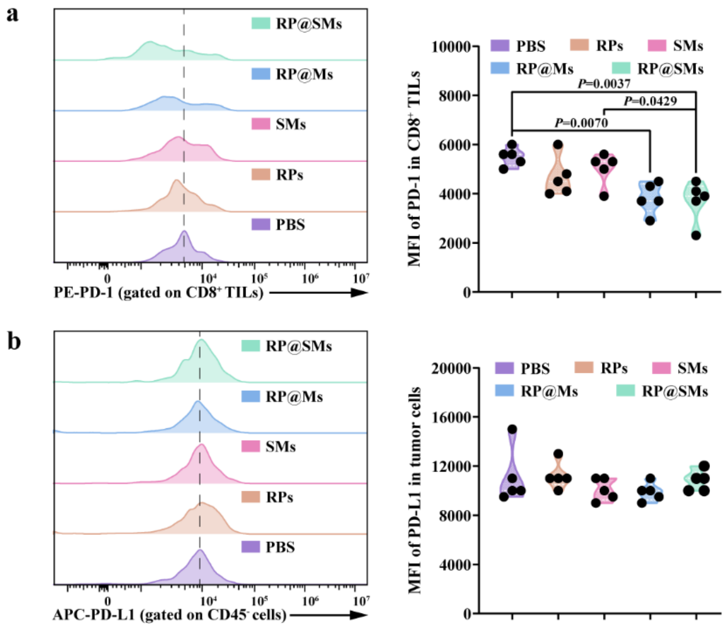


**Fig. S21 Effects of RP@SMs nanovaccine on PD-L1 expression on tumor cells and PD-1 expression on $CD8^+$ tumor-infiltrating lymphocytes. a**, Representative flow cytometry images and quantification of PD-1 expression on $CD8^+$ TILs ($n = 5$ mice per group). **b**, Representative flow cytometry images and quantification of PD-L1 expression on tumor cells ($n = 5$ mice per group). Data are presented as mean ± s.d. Statistical significance was determined using a one-way ANOVA with Tukey's multiple-comparisons test with a confidence interval of 95%.

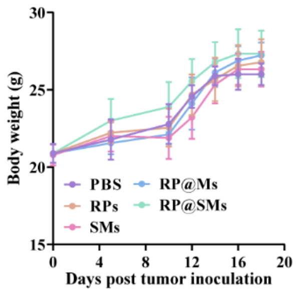


**Fig. S22 Effect of RP@SMs on mouse body weight.** Body weight curves of B16F10 tumor-bearing mice after different treatments ($n = 9$ mice per group).

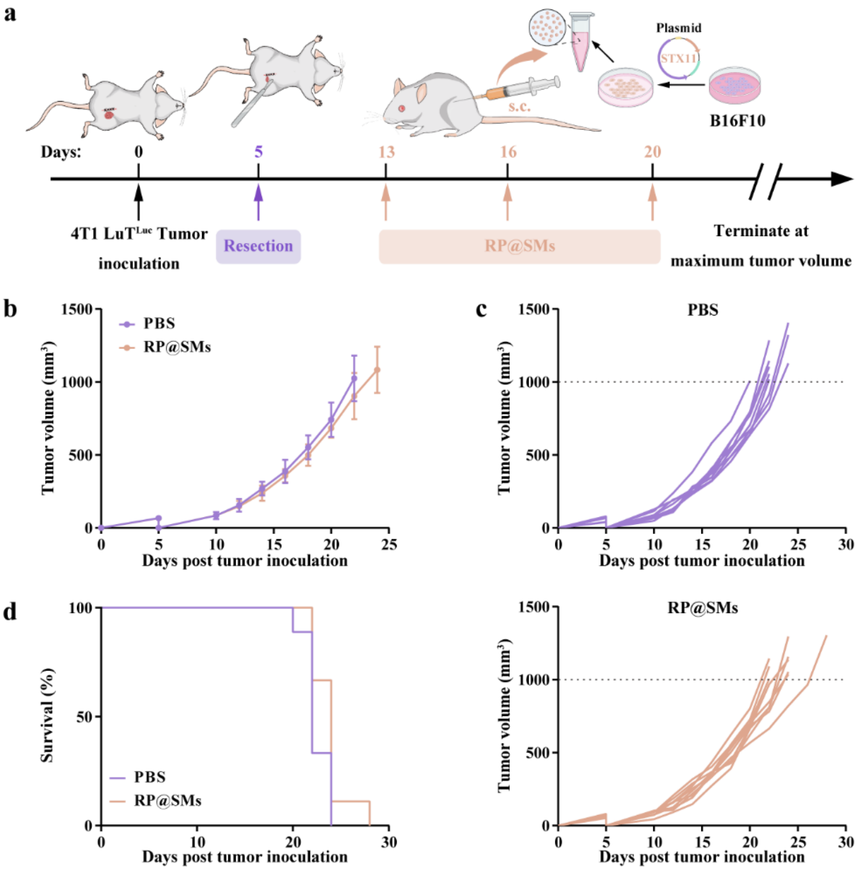


**Fig. S23 Lack of therapeutic efficacy of heterologous B16F10-derived RP@SMs in 4T1 LuT tumor-bearing mice. a**, Schematic illustration of the postoperative metastatic tumor recurrence model and vaccination schedule. **b**, Average tumor growth curves in each treatment group ($n = 9$ mice per group). **c**, Individual tumor growth trajectories for each mouse. **d**, Kaplan-Meier survival analysis of tumor-bearing mice ($n = 9$ mice per group). Data are presented as mean ± s.d. Statistical significance was determined using an unpaired two-sided t-test or a log-rank (Mantel-Cox) test with a confidence interval of 95%. Panel **a** created with BioRender.com.

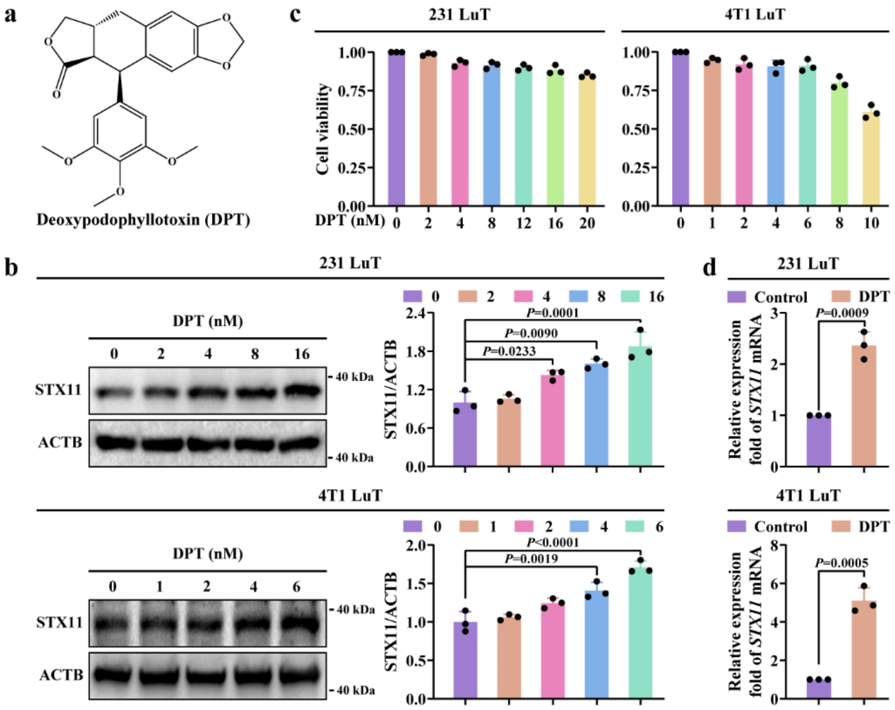


**Fig. S24 Effects of DPT on cell viability and STX11 protein expression in postoperative lung-metastatic TNBC cells. a**, Chemical structure of DPT. **b**, Protein levels of STX11 in 231 LuT and 4T1 LuT cells treated with various concentrations of DPT for 24 hours ($n = 3$ independent biological replicates). **c**, Cell viability assays for 231 LuT and 4T1 LuT cells after DPT treatment ($n = 3$ independent biological replicates). **d**, Gene expression of *STX11* in 231 LuT and 4T1 LuT cells after 24 h of DPT treatment, determined by RT-qPCR ($n = 3$ independent biological replicates). Data are presented as mean ± s.d. Statistical significance was determined using a one-way ANOVA with Tukey's multiple-comparisons test or an unpaired two-sided t-test with a confidence interval of 95%.

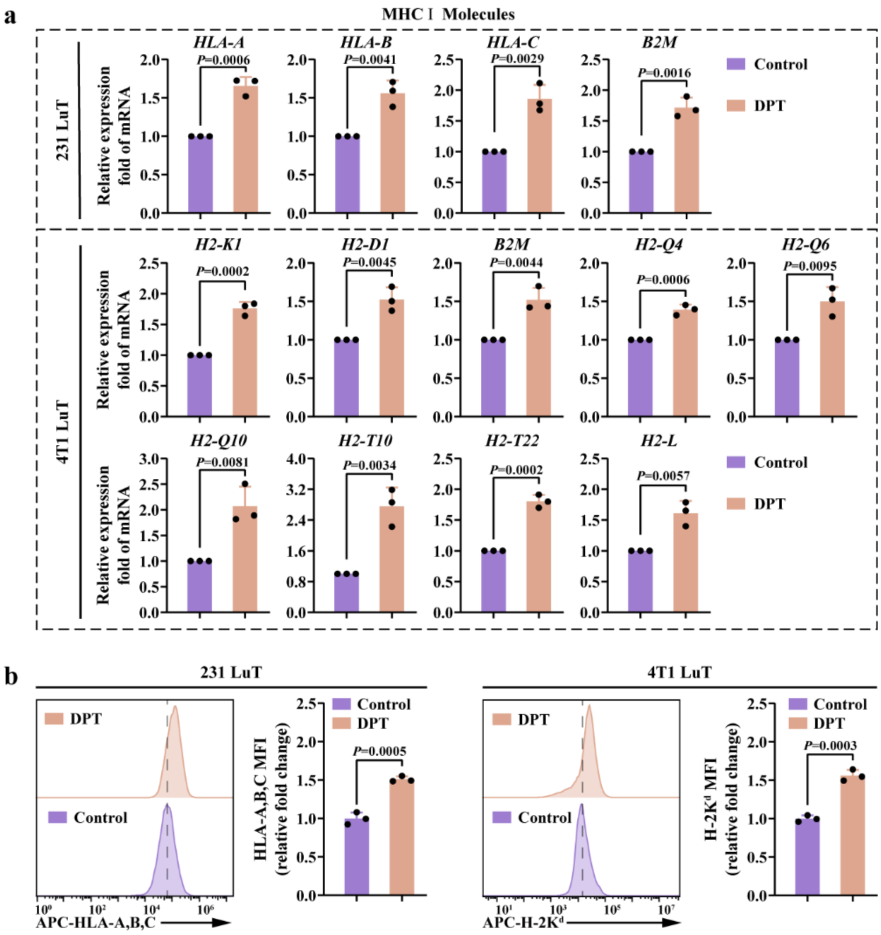


**Fig. S25 DPT enhances surface MHC I expression in postoperative lung-metastatic TNBC cells. a**,**b**, RT-qPCR analysis (**a**) and flow cytometry (**b**) of MHC I expression in 231 LuT and 4T1 LuT cells after 24-hour DPT treatment ($n = 3$ independent biological replicates). Data are presented as mean ± s.d. Statistical significance was determined using an unpaired two-sided t-test with a confidence interval of 95%.

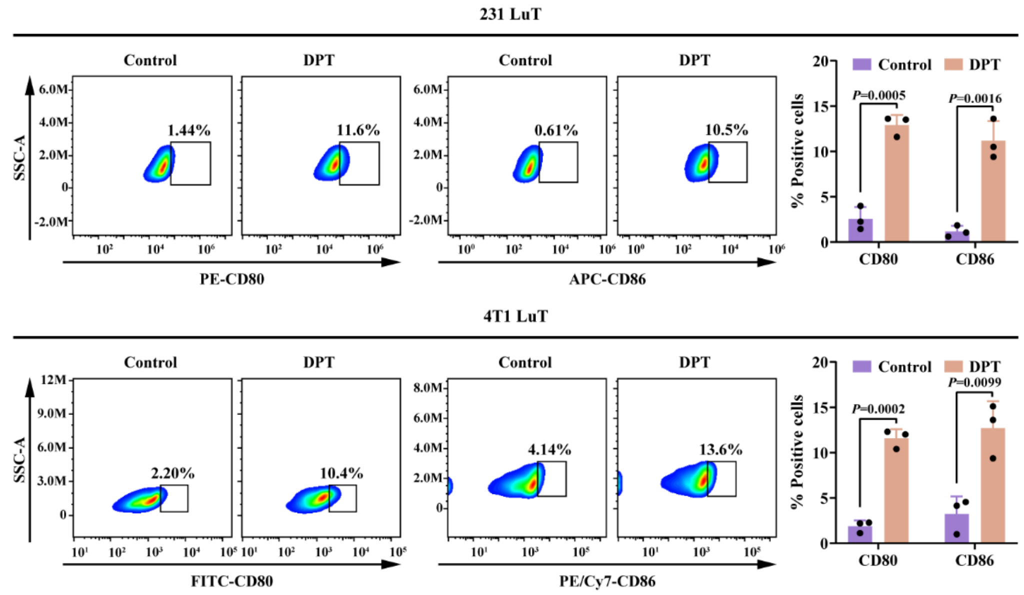


**Fig. S26 DPT enhances surface expression of CD80 and CD86 in postoperative lung-metastatic TNBC cells.** Flow cytometry analysis of CD80 and CD86 levels in 231 LuT and 4T1 LuT cells after 24-hour DPT treatment ($n = 3$ independent biological replicates). Data are presented as mean ± s.d. Statistical significance was determined using an unpaired two-sided t-test with a confidence interval of 95%.

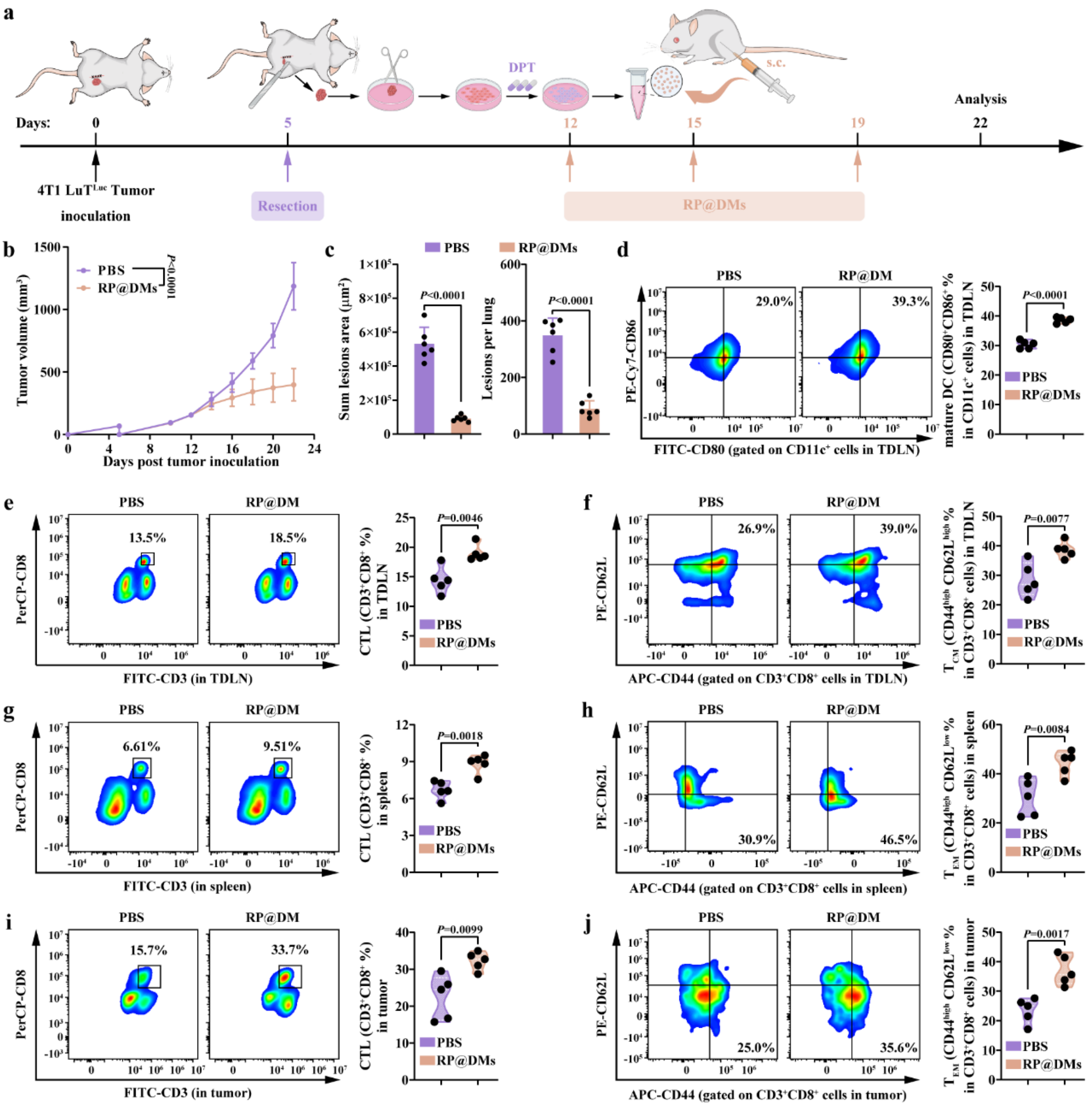


**Fig. S27 DPT-based RP@DMs personalized autologous vaccine for the treatment of postoperative metastatic TNBC. a**, Schematic of the RP@DMs personalized vaccine timeline in postoperative mice. **b**, Tumor growth curves across treatment groups (*n* = 10 mice per group). **c**, Quantification of lung metastases across treatment groups (*n* = 6 mice per group). **d**, Representative flow cytometry images and quantification of mature DCs in TDLNs (*n* = 5 mice per group). **e**, Representative flow cytometry images and quantification of CTLs in TDLNs (*n* = 5 mice per group). **f**, Representative flow cytometry images and quantification of $T_{CM}$ cells in TDLNs (*n* = 5 mice per group). **g**, Representative flow cytometry images and quantification of CTLs in the spleen (*n* = 5 mice per group). **h**, Representative flow cytometry images and quantification of $T_{EM}$ cells in the spleen (*n* = 5 mice per group). **i**, Representative flow

cytometry images and quantification of CTLs in tumor tissues ($n$ = 5 mice per group). **j**, Representative flow cytometry images and quantification of $T_{EM}$ cells in tumor tissues ($n$ = 5 mice per group). Data are presented as mean ± s.d. Statistical significance was determined using an unpaired two-sided t-test with a confidence interval of 95%. Panel **a** created with BioRender.com.

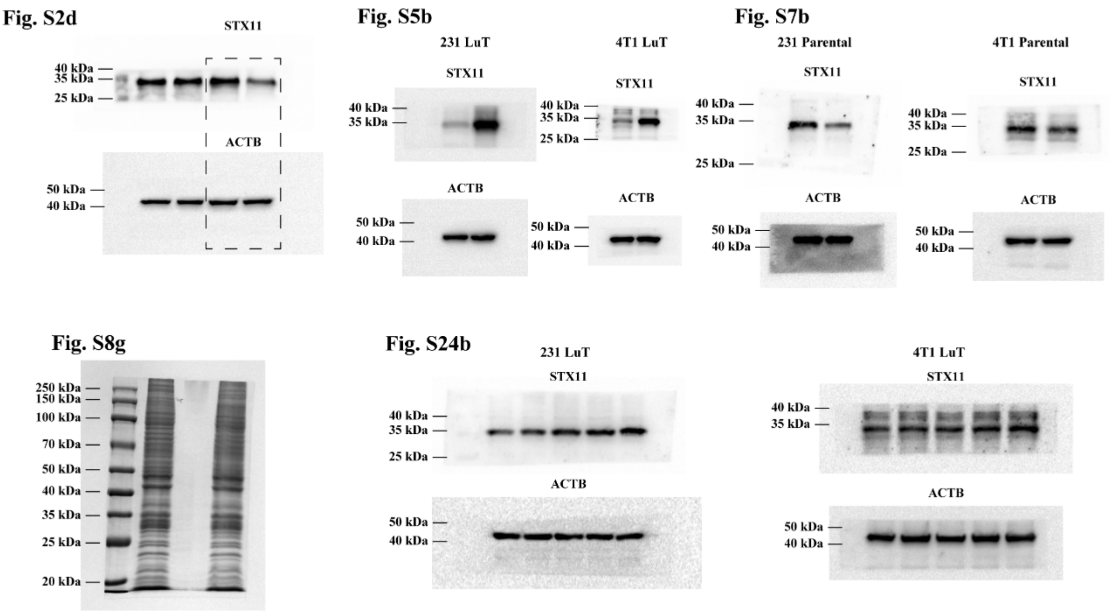


**Fig. S28 Unprocessed images of all gels and blots used in the Supplementary Figures.**

**Supplementary Table 1 Flow cytometry antibodies used in this study.**

| Antibodies | Manufacturers | Catalog No. | Clone No. | Dilution |
|---|---|---|---|---|
| anti-mouse CD16/32 | Biolegend | 101302 | 93 | 1:50 |
| FITC anti-mouse CD80 | Biolegend | 104705 | 16-10A1 | 1:50 |
| PE/Cyanine7 anti-mouse CD86 | Biolegend | 105013 | GL-1 | 1:20 |
| APC anti-mouse PD-L1 | Biolegend | 124311 | 10F.9G2 | 1:80 |
| eFluor 450 anti-mouse CD45 | eBioscience | 48-0451-80 | 30-F11 | 1:40 |
| APC anti-mouse/human CD44 | Biolegend | 103011 | IM7 | 1:80 |
| PerCP/Cyanine5.5 anti-mouse CD8a | Biolegend | 100734 | 53-6.7 | 1:20 |
| APC anti-mouse CD11c | Biolegend | 117310 | N418 | 1:80 |
| APC anti-mouse H-2K$^d$ | Biolegend | 116620 | SF1-1.1 | 1:80 |
| APC anti-human HLA-A,B,C | Biolegend | 311410 | W6/32 | 1:20 |
| PE/Cyanine7 anti-mouse IFN γ | eBioscience | 25-7311-41 | XMG1.2 | 1:20 |
| APC anti-mouse CD3 | Biolegend | 100235 | 17A2 | 1:40 |
| PE anti-mouse CD370 (CLEC9A, DNGR1) | Biolegend | 143503 | 7H11 | 1:80 |
| FITC anti-human HLA-A,B,C | Biolegend | 311404 | W6/32 | 1:20 |
| Super Bright™ 645 anti-mouse MHC Class II (I-A/I-E) | eBioscience | 64-5321-82 | M5/114.15.2 | 1:160 |
| FITC anti-mouse CD3 | Biolegend | 100204 | 17A2 | 1:50 |
| APC anti-mouse TNF α | Biolegend | 506307 | MP6-XT22 | 1:80 |
| PE anti-mouse PD-1 | eBioscience | 12-9985-81 | J43 | 1:40 |
| PE anti-mouse CD62L | Biolegend | 104407 | MEL-14 | 1:80 |
| PE anti-human CD80 | Biolegend | 305207 | 2D10 | 1:20 |
| APC anti-human CD86 | Biolegend | 374207 | BU63 | 1:20 |

**Supplementary Table 2 Western blot antibodies used in this study.**

| Antibodies | Manufacturers | Catalog No. | Dilution |
|---|---|---|---|
| STX11 | Proteintech | 13301-1-AP | 1:1000 |
| CD80 | Proteintech | 66406-1-lg | 1:2000 |
| CD86 | Proteintech | 13395-1-AP | 1:2000 |
| Na/K ATPase | Proteintech | 14418-1-AP | 1:5000 |
| MHC I | Santa Cruz | sc-59199 | 1:200 |
| ACTB | Proteintech | 66009-1-lg | 1:20000 |
| STX11 | Abcam | Ab216046 | 1:1000 |
| CCR7 | Abcam | ab32075 | 1:500 |
| Goat anti-Rat IgG HRP | Solarbio | SE132 | 1:200 |
| Goat anti-mouse IgG HRP | Abmart | M21001 | 1:5000 |
| Goat anti-Rabbit IgG HRP | Abmart | M21002 | 1:5000 |

**Supplementary Table 3 Sequences of RT-qPCR, shRNA and sgRNA primers.**

| Gene name | | Primer sequence (5’→3’) |
|---|---|---|
| | Human | |
| *HLA-A* | F | ATGTATGGCTGCGACGTGGG |
| | R | CTCCCACTTGCGCTTGGTGA |
| *HLA-B* | F | CTACAACCAGAGCGAGGCCG |
| | R | AATCCTTGCCGTCGTAGGCG |
| *HLA-C* | F | AGACCCAGGACACCGAGCTT |
| | R | CCATGATGGGGATGGTGGGC |
| *B2M* | F | GAGGCTATCCAGCGTACTCCA |
| | R | CGGCAGGCATACTCATCTTTT |
| *STX11* | F | AACTTGCTGGCCGACGTGAA |
| | R | CCTGCTTCTCCACCAGCACC |
| *GAPDH* | F | CCATGGAGAAGGCTGGGG |
| | R | CAAAGTTGTCATGGATGACC |
| shSTX11 | F | TTCTGGACTTGTCCAAGCAAT |
| | R | ATTGCTTGGACAAGTCCAGAA |
| | Mouse | |
| *H2-K1* | F | ACCAGCAGTACGCCTACGA |
| | R | AACCAGAACAGCAACGGTCG |
| *H2-D1* | F | AAGCCAAGGGCCAAGAGCAG |
| | R | GCGGCCTTCATAGGCGAACT |
| *B2M* | F | TTCTGGTGCTTGTCTCACTGA |
| | R | CAGTATGTTCGGCTTCCCATTC |
| *H2-Q4* | F | ATGGCGTCAACAATGCTGC |
| | R | GGGACACGGAGGTGTAGAA |
| *H2-Q10* | F | ACGTGGCGGCGATTATCAC |
| | R | AGGTAGGCCCTGTAATACTCTG |
| *H2-T10* | F | GGCTCGCAGACCCATGAAG |

| | | |
|---|---|---|
| | R | GGCCTAGACAAGTTTTACAGGAC |
| *H2-T22* | F | TCCCTTTGGGTTCACACTCG |
| | R | AGTCGTCCATGCTCTTGTTGT |
| *H2-L* | F | GATGCAGAGCATTACAGGGC |
| | R | GCCAGGTCAGGGCAATGTC |
| *STX11* | F | TGTTCGAGCAGGGCAAGTGG |
| | R | GCGTGTCCTCCTGCTTCTCC |
| *CD80* | F | GTCCTTTCAGACCGGGGCAC |
| | R | GGGAAACCCCCGGAAGCAAA |
| *CD86* | F | CTGCATATCTGCCGTGCCCA |
| | R | CGGCCCAGGTACTTGGCATT |
| *H2-Aa* | F | GTTCCCCAAGTCCCCTGTGC |
| | R | ACCGTCTGCGACTGACTTGC |
| *CD40* | F | GGTGTCTTTGCCTCGGCTGT |
| | R | TGCAGTGGCTTGTCAGTCGG |
| *CLEC9A* | F | CTGCAGCCCTTGTCCACACA |
| | R | GCCGGCTGATCGCTGTCTTT |
| *LY75* | F | AAGGCTGGCACACTTTCCCC |
| | R | AACCAACGCTGCCCACTGAA |
| *GAPDH* | F | AGGTCGGTGTGAACGGATTTG |
| | R | GGGGTCGTTGATGGCAACA |
| *STX11-sgRNA1* | F | CACCGACCCTCGATTACACGGGCG |
| | R | AAACCGCCCGTGTAATCGAGGGTC |
| *STX11-sgRNA2* | F | CACCGTGACTTTGACGCTCCTCGGG |
| | R | AAACCCCGAGGAGCGTCAAAGTCAC |
| *shSTX11* | F | CGCTGAACGTCATCGAGCTTA |
| | R | TAAGCTCGATGACGTTCAGCG |
