# Supplementary material for: Biomimetic vesicles engineered from modified tumour cells act as personalized vaccines for post-surgical cancer immunotherapy
Source: Nat Nanotechnol. 2026 Jan 29;21(3):443–54. doi: 10.1038/s41565-025-02113-w (PMC13017505; doi:10.1038/s41565-025-02113-w)
Supplement: Supplementary file 2 — Reporting Summary [file 41565_2025_2113_MOESM2_ESM.pdf]

nature portfolio

Corresponding author(s): Chao Zhang, Lingyi Kong

Last updated by author(s): Dec 3, 2025

Reporting Summary

Nature Portfolio wishes to improve the reproducibility of the work that we publish. This form provides structure for consistency and transparency in reporting. For further information on Nature Portfolio policies, see our Editorial Policies and the Editorial Policy Checklist.

Statistics

For all statistical analyses, confirm that the following items are present in the figure legend, table legend, main text, or Methods section.

n/a	Confirmed	
☐	☒	The exact sample size (*n*) for each experimental group/condition, given as a discrete number and unit of measurement
☐	☒	A statement on whether measurements were taken from distinct samples or whether the same sample was measured repeatedly
☐	☒	The statistical test(s) used AND whether they are one- or two-sided<br>*Only common tests should be described solely by name; describe more complex techniques in the Methods section.*
☐	☒	A description of all covariates tested
☐	☒	A description of any assumptions or corrections, such as tests of normality and adjustment for multiple comparisons
☐	☒	A full description of the statistical parameters including central tendency (e.g. means) or other basic estimates (e.g. regression coefficient) AND variation (e.g. standard deviation) or associated estimates of uncertainty (e.g. confidence intervals)
☐	☒	For null hypothesis testing, the test statistic (e.g. *F*, *t*, *r*) with confidence intervals, effect sizes, degrees of freedom and *P* value noted<br>*Give P values as exact values whenever suitable.*
☒	☐	For Bayesian analysis, information on the choice of priors and Markov chain Monte Carlo settings
☒	☐	For hierarchical and complex designs, identification of the appropriate level for tests and full reporting of outcomes
☐	☒	Estimates of effect sizes (e.g. Cohen's *d*, Pearson's *r*), indicating how they were calculated

Our web collection on statistics for biologists contains articles on many of the points above.

Software and code

Policy information about availability of computer code

Data collection	Flow cytometric data were collected using a CytoFLEX S flow cytometer (Beckman Coulter, USA).<br>The particle size, polydispersity index (PDI), and zeta potential of nanoparticles were measured using a Zetasizer Nano ZS90 (Malvern, UK).<br>In vivo fluorescence imaging of mice was performed with a Maestro™ in vivo imaging system (PerkinElmer, Waltham, MA, USA).<br>Nanoparticle morphology was characterized using transmission electron microscopy (HT7700, Hitachi, Tokyo, Japan).<br>Confocal laser scanning microscopy (LSM 800, Carl Zeiss, Germany) was used to visualize nanoparticle uptake and cellular localization.
Data analysis	Flow cytometry data were analyzed using FlowJo v10.9 (BD Biosciences).<br>Statistical analyses were performed using GraphPad Prism v8.0.2.<br>Fluorescence imaging data were analyzed using IVIS Living Image Software (PerkinElmer).<br>All statistical tests, including definitions of significance and error bars, are specified in the figure legends.

For manuscripts utilizing custom algorithms or software that are central to the research but not yet described in published literature, software must be made available to editors and reviewers. We strongly encourage code deposition in a community repository (e.g. GitHub). See the Nature Portfolio guidelines for submitting code & software for further information.

Data

Policy information about availability of data

All manuscripts must include a data availability statement. This statement should provide the following information, where applicable:

- Accession codes, unique identifiers, or web links for publicly available datasets
- A description of any restrictions on data availability
- For clinical datasets or third party data, please ensure that the statement adheres to our policy

Transcriptomic analyses and clinical information data from TCGA and GTEx databases (http://www.sangerbox.com/tool) and TIMER2.0 (http://timer.cistrome.org/). The raw data of single RNA sequencing can be found on the Gene Expression Omnibus at accession number (GSE199219). The source data generated in this study are provided in the Supplementary Information/Source data file.

Research involving human participants, their data, or biological material

Policy information about studies with human participants or human data. See also policy information about sex, gender (identity/presentation), and sexual orientation and race, ethnicity and racism.

Reporting on sex and gender	This study did not involve human participants.
Reporting on race, ethnicity, or other socially relevant groupings	This study did not involve human participants.
Population characteristics	This study did not involve human participants.
Recruitment	This study did not involve human participants.
Ethics oversight	This study did not involve human participants.

Note that full information on the approval of the study protocol must also be provided in the manuscript.

Field-specific reporting

Please select the one below that is the best fit for your research. If you are not sure, read the appropriate sections before making your selection.

☒ Life sciences ☐ Behavioural & social sciences ☐ Ecological, evolutionary & environmental sciences

For a reference copy of the document with all sections, see nature.com/documents/nr-reporting-summary-flat.pdf

Life sciences study design

All studies must disclose on these points even when the disclosure is negative.

Sample size	Sample sizes for definitive in vitro experiments were determined by power analysis based on expected effect sizes from prior literature and our pilot studies, ensuring adequate statistical power. For these experiments, a minimum of three biologically independent replicates ($n \geq 3$) were used per condition. For in vivo experiments, group sizes were selected based on variability estimates from previous similar models and a power analysis, while strictly adhering to the ethical 4R principles to minimize animal use. All key findings presented in the figures are derived from these statistically robust sample sizes. It is noted that in a limited number of preliminary validation experiments, a sample size of $n = 2$ was used initially to confirm trend consistency. However, the definitive results and conclusions for all reported assays are exclusively supported by independent experiments with a sample size of $n \geq 3$, as detailed in the figure legends. Throughout the manuscript, the "n" value represents biologically independent samples or independent experimental replicates, as explicitly specified in each figure legend.
Data exclusions	No data points or samples were excluded from any analyses.
Replication	All key experiments were independently repeated at least three times, and the results were reproducible. Material preparation, processing, and characterization were each performed in triplicate. Both in vitro and in vivo experiments were independently repeated at least three times to confirm the robustness of observed trends before inclusion in the final analyses.
Randomization	All animals and samples were randomly assigned to experimental groups. For in vivo studies, randomization was performed before treatment allocation to minimize bias.
Blinding	All surgical operations, sample processing, and data acquisition were performed by investigators blinded to group allocation. Data analysis was conducted without prior knowledge of treatment identity.

Reporting for specific materials, systems and methods

We require information from authors about some types of materials, experimental systems and methods used in many studies. Here, indicate whether each material, system or method listed is relevant to your study. If you are not sure if a list item applies to your research, read the appropriate section before selecting a response.

Materials & experimental systems

n/a	Involved in the study
☐	☒ Antibodies
☐	☒ Eukaryotic cell lines
☒	☐ Palaeontology and archaeology
☐	☒ Animals and other organisms
☒	☐ Clinical data
☒	☐ Dual use research of concern
☒	☐ Plants

Methods

n/a	Involved in the study
☒	☐ ChIP-seq
☐	☒ Flow cytometry
☒	☐ MRI-based neuroimaging

Antibodies

Antibodies used

Anti-mouse CD16/32 (Biolegend, Cat# 101302, clone 93, 1:50 dilution)
FITC anti-mouse CD80 (Biolegend, Cat# 104705, clone 16-10A1, 1:50 dilution)
PE/Cyanine7 anti-mouse CD86 (Biolegend, Cat# 105013, clone GL-1, 1:20 dilution)
APC anti-mouse PD-L1 (Biolegend, Cat# 124311, clone 10F.9G2, 1:80 dilution)
eFluor 450 anti-mouse CD45 (eBioscience, Cat# 48-0451-80, clone 30-F11, 1:40 dilution)
APC anti-mouse/human CD44 (Biolegend, Cat# 103011, clone IM7, 1:80 dilution)
PerCP/Cyanine5.5 anti-mouse CD8a (Biolegend, Cat# 100734, clone 53-6.7, 1:20 dilution)
APC anti-mouse CD11c (Biolegend, Cat# 117310, clone N418, 1:80 dilution)
APC anti-mouse H-2Kd (Biolegend, Cat# 116620, clone SF1-1.1, 1:80 dilution)
APC anti-human HLA-A,B,C (Biolegend, Cat# 311410, clone W6/32, 1:20 dilution)
PE/Cyanine7 anti-mouse IFN γ (eBioscience, Cat# 25-7311-41, clone XMG1.2, 1:20 dilution)
APC anti-mouse CD3 (Biolegend, Cat# 100235, clone 17A2, 1:40 dilution)
PE anti-mouse CD370 (CLEC9A, DNGR1) (Biolegend, Cat# 143503, clone 7H11, 1:80 dilution)
FITC anti-human HLA-A,B,C (Biolegend, Cat# 311404, clone W6/32, 1:20 dilution)
Super Bright™ 645 anti- mouse MHC Class II (I-A/I-E) (eBioscience, Cat# 64-5321-82, clone M5/114.15.2, 1:160 dilution)
FITC anti-mouse CD3 (Biolegend, Cat# 100204, clone 17A2, 1:50 dilution)
APC anti-mouse TNF α (Biolegend, Cat# 506307, clone MP6-XT22, 1:80 dilution)
PE anti-mouse PD-1 (eBioscience, Cat# 12-9985-81, clone J43, 1:40 dilution)
PE anti-mouse CD62L (Biolegend, Cat# 104407, clone MEL-14, 1:80 dilution)
PE anti-human CD80 (Biolegend, Cat# 305207, clone 2D10, 1:20 dilution)
APC anti-human CD86 (Biolegend, Cat# 374207, clone BU63, 1:20 dilution)
Anti-STX11 antibody (Proteintech, Cat# 13301-1-AP, 1:1000 dilution)
Anti-CD80 antibody (Proteintech, Cat# 66406-1-lg, 1:2000 dilution)
Anti-CD86 antibody (Proteintech, Cat# 13395-1-AP, 1:2000 dilution)
Anti-Na/K ATPase antibody (Proteintech, Cat# 14418-1-AP, 1:5000 dilution)
Anti-MHC I antibody (Santa Cruz, Cat# sc-59199, 1:200 dilution)
Anti-ACTB antibody (Proteintech, Cat# 66009-1-lg, 1:20000 dilution)
Anti-STX11 antibody (Abcam, Cat# Ab216046, 1:1000 dilution)
Anti-CCR7 antibody (Abcam, Cat# ab32075, 1:500 dilution)

Validation

Positive and negative controls of specific-binding (for each of the florescent labeled targets) were included in each experiment. Antibodies were used at the dilutions recommended by the manufacturer and the statements can be found on the manufactures' websites as follow:
Anti-mouse CD16/32 (https://www.biolegend.com/en-us/products/purified-anti-mouse-cd16-32-antibody-190)
FITC anti-mouse CD80 (https://www.biolegend.com/en-us/products/fitc-anti-mouse-cd80-antibody-41)
PE/Cyanine7 anti-mouse CD86 (https://www.biolegend.com/en-us/products/pe-cyanine7-anti-mouse-cd86-antibody-3046)
APC anti-mouse PD-L1 (https://www.biolegend.com/en-us/products/apc-anti-mouse-cd274-b7-h1-pd-l1-antibody-6655)
eFluor 450 anti-mouse CD45 (https://www.thermofisher.cn/cn/zh/antibody/product/CD45-Antibody-clone-30-F11-Monoclonal/48-0451-80)
APC anti-mouse/human CD44 (https://www.biolegend.com/en-us/products/apc-anti-mouse-human-cd44-antibody-312)
PerCP/Cyanine5.5 anti-mouse CD8a (https://www.biolegend.com/en-us/products/percp-cyanine5-5-anti-mouse-cd8a-antibody-4255)
APC anti-mouse CD11c (https://www.biolegend.com/en-us/products/apc-anti-mouse-cd11c-antibody-1813)
APC anti-mouse H-2Kd (https://www.biolegend.com/en-us/products/apc-anti-mouse-h-2kd-antibody-6845)
APC anti-human HLA-A,B,C (https://www.biolegend.com/en-us/products/apc-anti-human-hla-a-b-c-antibody-1870)
PE/Cyanine7 anti-mouse IFN γ (https://www.thermofisher.cn/cn/zh/antibody/product/IFN-gamma-Antibody-clone-XMG1-2-Monoclonal/25-7311-41)
APC anti-mouse CD3 (https://www.biolegend.com/en-us/products/apc-anti-mouse-cd3-antibody-8055)
PE anti-mouse CD370 (CLEC9A, DNGR1) (https://www.biolegend.com/en-us/products/pe-anti-mouse-cd370-clec9a-dngr1-antibody-7689)
FITC anti-human HLA-A,B,C (https://www.biolegend.com/en-us/products/fitc-anti-human-hla-a-b-c-antibody-1871)
Super Bright™ 645 anti- mouse MHC Class II (I-A/I-E) (https://www.thermofisher.cn/cn/zh/antibody/product/MHC-Class-II-I-A-I-E-Antibody-clone-M5-114-15-2-Monoclonal/64-5321-82)
FITC anti-mouse CD3 (https://www.biolegend.com/en-us/products/fitc-anti-mouse-cd3-antibody-45)

APC anti-mouse TNF α (https://www.biolegend.com/en-us/products/apc-anti-mouse-tnf-alpha-antibody-975)
PE anti-mouse PD-1 (https://www.thermofisher.cn/cn/zh/antibody/product/CD279-PD-1-Antibody-clone-J43-Monoclonal/12-9985-82?imageId=90157)
PE anti-mouse CD62L (https://www.biolegend.com/en-us/products/pe-anti-mouse-cd62l-antibody-386)
PE anti-human CD80 (https://www.biolegend.com/en-us/products/pe-anti-human-cd80-antibody-554)
APC anti-human CD86 (https://www.biolegend.com/en-us/products/apc-anti-human-cd86-antibody-15297)
Anti-STX11 antibody (https://www.ptgcn.com/products/STX11-Antibody-13301-1-AP.htm)
Anti-CD80 antibody (https://www.ptgcn.com/products/B7-1-Antibody-66406-1-Ig.htm)
Anti-CD86 antibody (https://www.ptgcn.com/products/CD86-Antibody-13395-1-AP.htm)
Anti-Na/K ATPase antibody (https://www.ptgcn.com/products/ATP1A1-Antibody-14418-1-AP.htm)
Anti-MHC I antibody (https://www.scbt.com/zh/p/mhc-class-i-antibody-er-hr52)
Anti-ACTB antibody (https://www.ptgcn.com/products/Pan-Actin-Antibody-66009-1-Ig.htm)
Anti-STX11 antibody (https://www.abcam.cn/products/primary-antibodies/stx11-antibody-ab216046)
Anti-CCR7 antibody (https://www.abcam.cn/products/primary-antibodies/ccr7-antibody-e75-ab32075)

Eukaryotic cell lines

Policy information about cell lines and Sex and Gender in Research

Cell line source(s)	Human breast cancer cell line MDA-MB-231 (TCHu227), mouse breast cancer cell line 4T1, (TCM32) and mouse melanoma cell line B16F10 (TCM36) were sourced from the Cell Bank of the Shanghai Institute of Life Sciences. 4T1 and B16F10 cells were cultured in RPMI 1640 medium, whereas MDA-MB-231 cells were maintained in DMEM, both supplemented with 10% fetal bovine serum and 1% penicillin-streptomycin under standard conditions (37°C, 5% CO2).
Authentication	The cell lines were authenticated using Short Tandem Repeat (STR) analysis.
Mycoplasma contamination	Regular mycoplasma testing confirmed the absence of contamination.
Commonly misidentified lines (See ICLAC register)	No commonly misidentified cell lines were used.

Animals and other research organisms

Policy information about studies involving animals; ARRIVE guidelines recommended for reporting animal research, and Sex and Gender in Research

Laboratory animals	Six- to eight-week-old C57BL/6 male mice, BALB/c female mice, and NCG (NOD/ShiLtJGpt-Prkdcem26Cd52Il2rgem26Cd22/Gpt) female mice were purchased from GemPharmatech Co., Ltd. (Nanjing, China) and maintained under specific pathogen-free (SPF) conditions. CD11c-DTR female mice were obtained from Aniphe Biolaboratory Inc. (Nanjing, China) and housed under identical conditions.<br>All animal studies were approved by the Animal Ethics Committee of China Pharmaceutical University and conducted in compliance with institutional and national guidelines for animal care and use.<br>Animals were housed six per cage under a 12 h light/dark cycle (lights on at 08:00), at a controlled temperature (~25 °C) and humidity (50–80%), with ad libitum access to standard laboratory chow and tap water throughout the study.
Wild animals	This study did not involve the use of any wild animals.
Reporting on sex	Six- to eight-week-old BALB/c, NCG, and CD11c-DTR female mice were used for breast cancer models.<br>Six- to eight-week-old C57BL/6 male mice were used for melanoma models.
Field-collected samples	No field-collected samples were used in this study.
Ethics oversight	All animal studies were reviewed and approved by the Animal Ethics Committee of China Pharmaceutical University (Approval No. 2021-01-021).

Note that full information on the approval of the study protocol must also be provided in the manuscript.

Plants

Seed stocks	N/A
Novel plant genotypes	N/A
Authentication	N/A

Flow Cytometry

Plots

Confirm that:

☒ The axis labels state the marker and fluorochrome used (e.g. CD4-FITC).

☒ The axis scales are clearly visible. Include numbers along axes only for bottom left plot of group (a 'group' is an analysis of identical markers).

☒ All plots are contour plots with outliers or pseudocolor plots.

☒ A numerical value for number of cells or percentage (with statistics) is provided.

Methodology

Sample preparation	BMDCs were isolated by flushing the femurs and tibias of BALB/c mice with serum-free RPMI 1640 medium. After removing red blood cells with red blood cell (RBC) lysis buffer, the remaining cells were centrifuged to collect the cell pellet, which was then cultured in RPMI 1640 medium containing 20 ng/mL GM-CSF (78017.2, STEMCELL Technologies) and 10 ng/mL IL-4 (78047.2, STEMCELL Technologies) to promote DC growth and differentiation. The medium was refreshed every two days, and on the sixth day, adherent BMDCs were collected for further experimentation.<br>The tumor tissues were finely minced and digested with Type IV collagenase, hyaluronidase, and DNase I (Sigma-Aldrich) at 37°C for 1 hour. The resulting tumor homogenates were centrifuged at 350 g for 5 minutes, and red blood cells were lysed using RBC lysis buffer. The remaining cells were resuspended in PBS and passed through a 70 µm cell strainer to obtain a single-cell suspension. Spleens and tumor-draining lymph nodes (TDLNs) were mechanically disrupted, followed by RBC lysis and filtration through a 70 µm cell strainer to prepare single-cell suspensions. All cell suspensions were maintained in PBS containing 2% FBS.<br>For cell surface marker analysis, cells were first blocked with CD16/32 antibody for 15 minutes to prevent nonspecific binding. The cells were then incubated at room temperature for 15 minutes with various fluorescent antibodies, including APC-CD11c, FITC-CD80, PE/Cyanine7-CD86, FITC-CD3, PerCP/Cyanine5.5-CD8, APC-CD44, PE-CD62L, eFluor 450-CD45, PE-PD-1, and APC-PD-L1, following the manufacturer's instructions. Subsequently, cells were stained with SYTOXTM AADvancedTM Dead Cell Stain (S10349, ThermoFisher) at 4°C for 15 minutes.<br>For intracellular cytokine staining, surface-stained cells were subsequently labeled with Fixable Viability Dye eFluor™ 450 (65-0863-14, eBioscience) in the dark at 4 °C for 15 min, followed by fixation, permeabilization, and incubation with PE/Cy7-IFNγ and APC-TNFα antibodies for 15 min. After washing, flow cytometry was performed using a CytoFLEX S flow cytometer (Beckman Coulter).
Instrument	CytoFLEX S flow cytometer
Software	Flow cytometric data were analyzed with FlowJo v10.9.
Cell population abundance	For each sample, 10,000 events were collected after gating.
Gating strategy	Gating was first performed on FSC-H/SSC-H to identify the main cell population, followed by singlet gating on SSC-H versus SSC-A. Detailed gating strategies are provided in Supplementary Figures 12, 13, 16–18.

☒ Tick this box to confirm that a figure exemplifying the gating strategy is provided in the Supplementary Information.
