# Supplementary material for: Biomimetic vesicles engineered from modified tumour cells act as personalized vaccines for post-surgical cancer immunotherapy
Source: Nat Nanotechnol. 2026 Jan 29;21(3):443–54. doi: 10.1038/s41565-025-02113-w (PMC13017505; doi:10.1038/s41565-025-02113-w)
Supplement: Supplementary file 4 — STR profiling analysis of cell lines. [file 41565_2025_2113_MOESM4_ESM.pdf]

**Supplementary Table** STR profiling analysis of cell lines.

<table>
<tr><th colspan="2">Sample</th><th colspan="4">MDA-MB-231</th></tr>
<tr><th colspan="6">Method and Procedure</th></tr>
<tr><td colspan="6">Sample DNA was extracted by Microread Genomic DNA Kit.<br>PCR was amplified with STR Multi-amplification Kit (MicroreaderTM21 ID System).<br>PCR products were assayed with ABI 3730xl DNA Analyzer (Applied Biosystems®).<br>Data were analyzed using GeneMapperID-X software and then compared with the ATCC and DSMZ databases for reference matching.</td></tr>
<tr><th colspan="6">Results</th></tr>
<tr><td>Locus</td><td>Results</td><td>Locus</td><td>Results</td><td>Locus</td><td>Results</td></tr>
<tr><td>D5S818</td><td>12</td><td>D16S539</td><td>12</td><td>Amelogenin</td><td>X</td></tr>
<tr><td>D13S317</td><td>13</td><td>vWA</td><td>15, 18</td><td>TPOX</td><td>8, 9</td></tr>
<tr><td>D7S820</td><td>8, 9</td><td>ITH01</td><td>7, 9.3</td><td>CSF1PO</td><td>12, 13</td></tr>
<tr><th colspan="6">Conclusion</th></tr>
<tr><td colspan="6">The above results were consistent with the DNA profiles reported by ATCC and DSMZ, and indicated no other human cell lines contamination.</td></tr>
<tr><td colspan="6"></td></tr>
<tr><th colspan="2">Sample</th><th colspan="4">4T1</th></tr>
<tr><th colspan="6">Results</th></tr>
<tr><td>Locus</td><td>Results</td><td>Locus</td><td>Results</td><td>Locus</td><td>Results</td></tr>
<tr><td>1-1</td><td>15, 16</td><td>6-7</td><td>12, 12</td><td>17-2</td><td>15, 15</td></tr>
<tr><td>1-2</td><td>17, 17</td><td>7-1</td><td>25.2, 25.2</td><td>18-3</td><td>18, 19</td></tr>
<tr><td>2-1</td><td>16, 17</td><td>8-1</td><td>13, 13</td><td>19-2</td><td>13, 13</td></tr>
<tr><td>3-2</td><td>14, 15</td><td>11-2</td><td>18, 19, 20</td><td>X-1</td><td>25, 25</td></tr>
<tr><td>4-2</td><td>21.3, 21.3</td><td>12-1</td><td>16, 16</td><td>TH01</td><td></td></tr>
<tr><td>5-5</td><td>14, 14</td><td>13-1</td><td>16.2, 16.2</td><td>D5S818</td><td></td></tr>
<tr><td>6-4</td><td>18, 18</td><td>15-3</td><td>22.3, 22.3</td><td></td><td></td></tr>
<tr><th colspan="6">Conclusion</th></tr>
<tr><td colspan="6">1) The tested cell is mouse cell line(s).</td></tr>
</table>

| | | | | | |
|---|---|---|---|---|---|
| 2) The submitted profile has a 97.96% match for the following mouse cell line(s) in ExPASy database (18 mouse STR loci plus 2 human STR loci): 4T1.<br>3) No cross-contamination of human cell line(s)has been identified in this mouse cell line(s). | | | | | |
| | | | | | |
| **Sample** | | **B16F10** | | | |
| **Results** | | | | | |
| Locus | Results | Locus | Results | Locus | Results |
| 1-1 | 17, 18, 19 | 6-7 | 15, 15 | 17-2 | 15, 16, 17 |
| 1-2 | 19, 20 | 7-1 | 26.2, 26.2 | 18-3 | 15, 16 |
| 2-1 | 16, 16 | 8-1 | 16, 17 | 19-2 | 13, 14 |
| 3-2 | 14, 15 | 11-2 | 16, 17 | X-1 | 28, 28 |
| 4-2 | 20.3, 21.3 | 12-1 | 17, 18 | TH01 | |
| 5-5 | 16, 20 | 13-1 | 17.1, 18.1 | D5S818 | |
| 6-4 | 18, 19 | 15-3 | 22.3, 23.3 | | |
| **Conclusion** | | | | | |
| 1) The tested cell is mouse cell line(s).<br>2) The submitted profile has a100% match for the following mouse cell line(s) in ExPASy database (18 mouse STR loci plus 2 human STR loci): B16F10.<br>3) No cross-contamination of human cell line(s)has been identified in this mouse cell line(s). | | | | | |
