# Supplementary figures and images for: Biomimetic vesicles engineered from modified tumour cells act as personalized vaccines for post-surgical cancer immunotherapy
Source: Nat Nanotechnol. 2026 Jan 29;21(3):443–54. doi: 10.1038/s41565-025-02113-w (PMC13017505; doi:10.1038/s41565-025-02113-w)

**Fig. 3d**

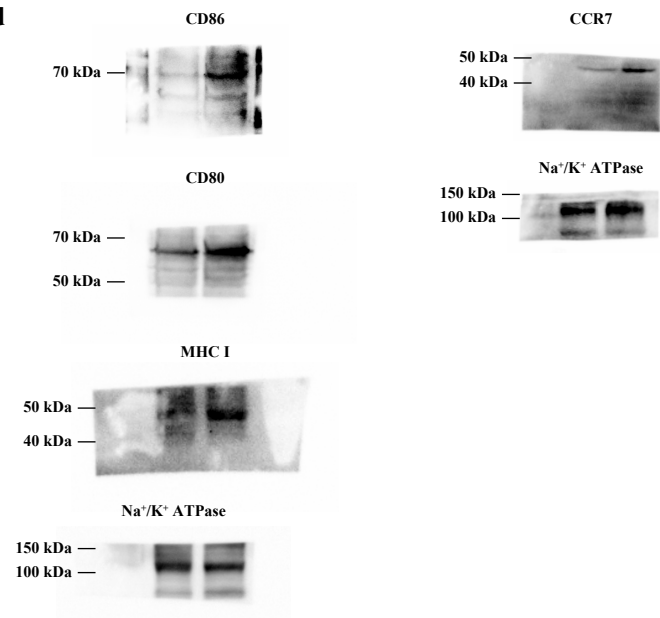

Supplement: Supplementary file 7 — Unprocessed western blots. [file 41565_2025_2113_MOESM7_ESM.pdf]

Extended Data Fig. 1c

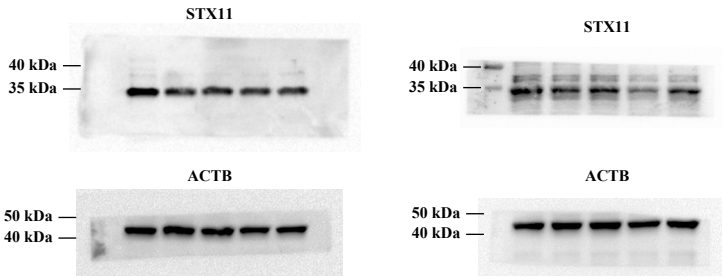

Supplement: Supplementary file 12 — Unprocessed western blots. [file 41565_2025_2113_MOESM12_ESM.pdf]
